# Critical raw material-free multi-principal alloy design for a net-zero future

**DOI:** 10.1038/s41598-025-87784-0

**Published:** 2025-01-24

**Authors:** Swati Singh, Mingwen Bai, Allan Matthews, Saurav Goel, Shrikrishna N. Joshi

**Affiliations:** 1 Department of Mechanical Engineering, Indian Institute of Technology Guwahati, Guwahati, 781039 India; 2 School of Mechanical Engineering, University of Leeds, Leeds, LS2 9JT UK; 3 Henry Royce Institute, The University of Manchester, Manchester, M13 9PL UK; 4 School of Engineering, London South Bank University, 103 Borough Road, London, SE1 0AA UK; 5 University of Petroleum and Energy Studies, Dehradun, 248007 India

**Keywords:** MPEAs, HEAs, Net zero, Machine learning, CRMs, Materials science, Nanoscience and technology

## Abstract

Refractory High-Entropy Alloys (RHEAs), such as NbMoTaW, MoNbTaVW, HfNbTaZr, Re_0.1_Hf_0.25_NbTaW_0.4_, Nb_40_Ti_25_Al_15_V_10_Ta_5_Hf_3_W_2_, Ti_***x***_NbMoTaW (x = 0, 0.25, 0.5, 0.75 and 1), and 3d transition metal HEAs such as Al_10.3_Co_17_Cr_7.5_Fe_9_Ni_48.6_Ti_5.8_Ta_0.6_Mo_0.8_W_0.4_ have demonstrated superior performance compared to traditional superalloys, particularly in high-temperature applications for engine components. However, the development of these alloys often depends on critical raw materials (CRMs) such as Ta, W, Nb, Hf, and others. The reliance on critical raw materials (CRMs) not only generates substantial emissions during recycling processes but also imposes considerable risks across global supply chains, hindering the pursuit of Net-zero ambitions. In this pioneering work, we unveil an inventive approach to inversely predict novel multicomponent alloy compositions, meticulously crafted to eliminate CRMs while achieving hardness levels comparable to those of CRM-containing multi-principal element alloys (MPEAs). A robust machine learning (ML) model was developed using a computational database of 3,608 entries, covering unary and binary materials from the Thermo-Calc 2024a software. Among various ML models, the Extra Trees Regressor (ETR) exhibited superior performance and was integrated with metaheuristic optimization techniques to identify novel MPEA compositions. The Cuckoo Search Optimization (CSO) method produced reduced-CRM MPEAs that closely matched Thermo-Calc predictions, with an error margin below ± 20%. To assess the efficacy of these reduced-CRM MPEAs, we compared the hardness of newly synthesized MPEA with CRM-containing counterparts reported in the literature, particularly those with high-risk critical raw materials like Niobium (Nb) and Tantalum (Ta). For example, the CoCrFeNb_0.309_Ni alloy, which includes CRMs Nb and Co exhibits a Vickers hardness of 480 HV. In contrast, our proposed composition, Ti_0.01111_NiFe_0.4_Cu_0.4_ achieves a comparable hardness of 488 HV without using a CRM. Our objective was not to develop high hardness alloy but to facilitate the development of reduced-CRM multi-principal element alloys (R-CRM-MPEAs). We validated our computational approach through the experimental synthesis of an FCC-phase alloy, Al_6.25_Cu_18.75_Fe_25_Co_25_Ni_25_. Thermo-Calc evaluation and ML model predictions of the Vickers hardness showed excellent agreement with the experimental hardness values, which lends credence to our approach. In conclusion, this study provides a robust framework for accelerating the discovery of novel R-CRM-MPEAs, effectively addressing challenges related to supply chain vulnerabilities, import dependence, and related environmental concerns.

**Supplementary Information:**

The online version contains supplementary material available at 10.1038/s41598-025-87784-0.

## Introduction

High entropy alloys (HEAs), also known as multi-principal element alloys (MPEAs) or complex concentrated alloys (CCAs), are solid solution alloys containing five or more elements in equiatomic or near equiatomic proportions (between 5% and 35% atomic concentration). The increase in configurational entropy of mixing elements overcomes the enthalpies of compound formation, inhibiting intermetallic formation, which stabilises the solid solution in a single phase with high configurational entropy^1,2^.

Cantor alloy (CrMnFeCoNi) was among the first reported MPEA in 2004 that showed exceptional properties in the race of CCAs^3^. However, Al_x_(CrFeCoNiCu) with varying Al concentration (x = 0 to 3) was also developed at the same time by Yeh^4^ and it was after this effort that the name high-entropy alloy (HEA) was coined. Since then, various CCAs/MPEAs/HEAs have emerged due to their exceptional mechanical properties over conventional alloys.

Various refractory HEAs (RHEAs) such as NbMoTaW^5^, Ti_x_NbMoTaW (x = 0, 0.25, 0.5, 0.75, 1)^5^ V_x_NbMoTa (x = 0.25, 0.5, 0.75, 1.0)^6^, Nb_40_Ti_25_Al_15_V_10_Ta_5_Hf_3_W_2_^7^, NbMoTaW(HfN)_*x*_ (*x* = 0, 0.3, 0.7, 1.0)^8^, MoNbTaVW^9^, HfNbTaZr^9^, Re_0.1_Hf_0.25_NbTaW_0.4_^10^ and some 3d transition metal HEAs such as Al_10.3_Co_17_Cr_7.5_Fe_9_Ni_48.6_Ti_5.8_Ta_0.6_Mo_0.8_W_0.4_^11^, Al_10.2_Co_16.9_Cr_7.4_Fe_8.9_Ni_47.9_Ti_5.8_Mo_0.9_Nb_1.2_W_0.4_C_0.4_^12^ have been developed for high-temperature applications in aerospace, gas turbine, and nuclear power plants. Other MPEAs such as CoCrFeNiTa_x_ (x = 0, 0.1, 0.2, 0.3, 0.4, 0.5, and 0.75)^13^, CoCrFeNiNb_x_ (x = 0, 0.103, 0.155, 0.206, 0.309 and 0.412)^14^ and CoCrFeNiNb_x_ (x = 0.1, 0.25, 0.5 and 0.8)^15^ have also shown a remarkable combination of high strength and ductility for their eutectic counterparts such as CoCrFeNiTa_0.4_ and CrFeCoNiNb_0.5_ respectively. However, it is noticeable that these alloys are reliant on the use of critical raw materials (CRMs) such as Ta, W, Nb and Hf^16^. The availability of Hf powder is extremely limited^17^. Rizzo et al.^16^ have alluded to the importance of having a flawless supply chain of raw materials to maintain a sustainable circular economy. Thus, the exigency of minimizing the use of CRMs to mitigate the excess of imports and reducing the need for excessive mining to accelerate the transition to net zero became the prime focus of this study.

**Fig. 1:**
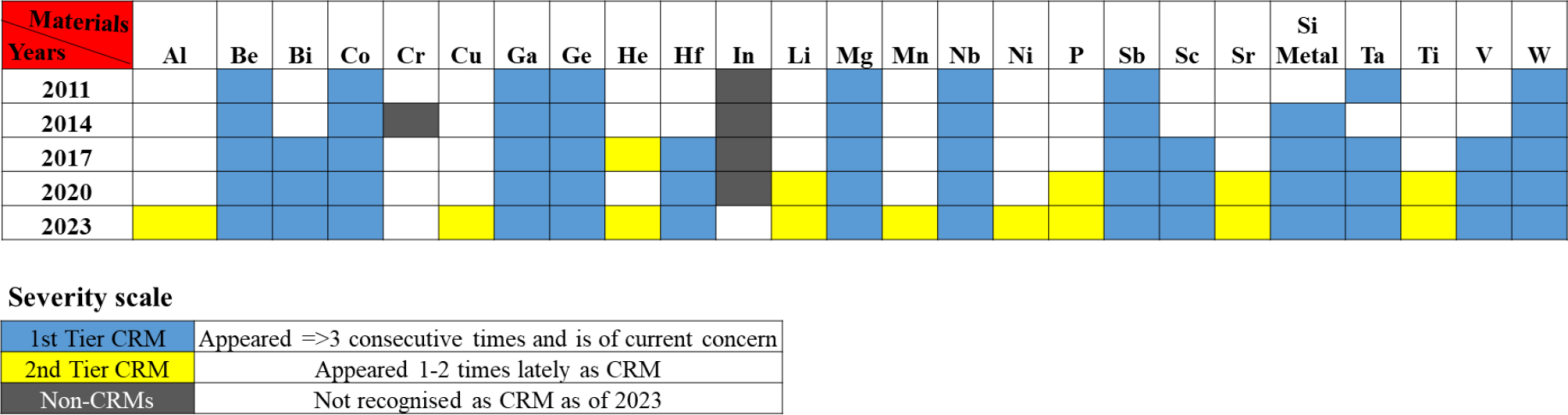
The severity scale of materials listed in the category of CRMs by the EU^18–22^.

The classification of CRM is an important distinction in the realization of this research. Accordingly, various CRMs identified in the past and present were tabulated and classified under three categories (see Fig. 1).

CRMs that appeared three or more times and remained a current concern were classed as 1st Tier. This includes elements such as Be, Bi, Co, Ga, Ge, Hf, Mg, Nb, Sb, Sc, Si Metal, Ta, V and W which are considered most critical in their usage. The 2nd Tier CRMs include elements such as Al, Cu, He, Li, Mn, Ni, P, Sr, and Ti, which have appeared recently as CRMs for more than once. Materials such as Cr and In are not considered CRMs as they were excluded from the 2023 list of CRMs and therefore are not considered as CRM in this study. An exception was made in the algorithm to include Co and V which are although classed as 1st Tier CRMs were not treated as critical. This is due to the ongoing advances made in the recovery and recycling methods and it is expected that the Co and V will become non-critical over time^23–26^.

A description of strategies adopted in recovering and recycling Co and V has been discussed in Section “Data collection using Thermo-Calc 2024a”. Henceforth, we considered Co and V as non-CRM for the current investigation and included them in the database for our current investigation. Accordingly, we report:

- Preparation of a fresh database reporting Vickers hardness of unary (pure) and binary-based compositions for non-CRM (Al, Cr, Cu, Fe, Ni, Ti, Mo, Mn, Sn, Zn, Zr) and CRM elements (Co and V- treated as non-critical in this study) using Thermo-Calc 2024a property model calculator based on CALPHAD approach. A total of 3,608 instances were recorded and no experimental data was incorporated to keep the database free from experimental uncertainty arising from the manufacturing process.
- Training and testing of various tree-based regression models: Decision Tree Regressor (DTR), Random Forest Regressor (RFR), AdaBoost Regressor (ABR), Gradient Boost Regressor (GBR), XGBoost Regressor (XGBR) and Extra Tree Regressor (ETR) based on the developed database and their performance evaluation based on various regression metrics such as coefficient of determination (R^2^_score), mean absolute error (MAE), mean squared error (MSE), root mean squared error (RMSE), and mean absolute percentage error (MAPE) for finding the robust regression model (best predictor) to correctly predict hardness of an unseen instance.
- Optimisation of the best regression model using metaheuristic optimisation techniques such as Particle Swarm Optimisation (PSO), Genetic Algorithm (GA), Ant Colony Optimisation (ACO), Cuckoo Search Optimisation (CSO) and Whale Optimisation Algorithm (WOA) in search of new multicomponent compositions to generate Reduced-CRM multi-principal element alloys (R-CRM-MPEAs) with target Vickers hardness values. Other optimization algorithms such as Artificial Bees Colony (ABC) and Simulated Annealing (SA) were also optimised for the same objective, but they both failed to generate the desired compositions.
- The ML-predicted hardness values were benchmarked to the corresponding predictions obtained from the Thermo-Calc calculator to test the robustness of the optimization models and compare the percentage error between ML and Thermo-Calc prediction of hardness value for the same compositions.
- A thorough evaluation was performed on experimentally synthesized CRM-laden MPEAs from the literature to benchmark the predicted R-CRM-MPEAs compositions generated in this study. This comparison aimed to demonstrate the feasibility of partially or fully replacing CRMs while preserving the hardness of the alloy.
- As a test case, Vickers hardness of a newly predicted composition (Al_6.25_Cu_18.75_Fe_25_Co_25_Ni_25_) developed by our group^27–29^ was measured experimentally and compared with the corresponding Thermo-Calc and ML predicted values successfully which lends credence to the computational prediction.

## Methodology

Most recent HEAs developed have been obtained by adjusting the composition percentages or substituting one element from an already established HEA. For example, various research articles built the new composition based on cantor alloy (CoCrFeMnNi) either by replacement of (CrFeCoNiCu^30^ and TiCrFeCoNi^31^), variation (CrMnFeCo_*x*_Ni and CrMnFeCoNi_*x*_ with *x* = 0–2^32^), or by addition (CrMnFeCoNiCu^3^, CrMnFeCoNiAl_*x*_^33^) of an element^2^. Additionally, reduction-based alloys have spawned lower-order systems such as binaries, ternaries and quaternary alloys- termed low and medium-entropy alloys^2^. Ten binaries, ten ternaries and five quaternaries’ compositions can be made from a cantor alloy. Among two of the ten possible binaries (FeNi and CoNi), five of the ten possible ternaries (CoFeNi, CrFeNi, FeMnNi, CoCrNi and CoMnNi), and three of the five possible quaternary (CoCrFeNi, CoFeMnNi and CoCrMnNi) are single phase FCC solid solution^34,35^. Interestingly, equiatomic CoCrNi medium entropy alloy shows better mechanical properties than the CoCrFeMnNi HEA, which demonstrates that configurational entropy (increasing the number of elements in an alloy) does not necessarily improve the mechanical property of an alloy^36^. Moreover, numerous research articles employed various state-of-the-art ML strategies in designing and developing novel MPEAs^37–40^.

Apart from these compositions, various MPEAs have been developed by utilising elements such as Al, Cu, Cr, Ti, V, W, Ta, Hf, Nb, Mo, Zn, Zr, Si extensively, while certain precious metal HEAs incorporate elements such as Ag, Pt, Au, Ru, Rh, Pd. However, many of these elements (Hf, Nb, Ta, Pt, Pd, Ru, Rh, W) have been marked as critical and have reached an alarming stage^41^. Consequently, when considering MPEAs comprised solely of non-CRMs elements, the available experimental data in the literature is scarce which limits the training and testing of the machine learning models. Therefore, we extracted a fresh database using Thermo-Calc 2024a software, which is based on a CALculation of PHAse Diagram (CALPHAD) approach. This database contains Vickers hardness values for unary (pure) and binary compositions of materials extracted from Thermo-Calc software using the TCHEA7 database. The work aimed to discover R-CRM-MPEAs compositions (from unary and binary composition databases) with mechanical properties comparable to CRM-laden MPEA compositions.

In recent years, CALPHAD has played a crucial role in designing transition alloys from a completely serendipitous process to a well-established method seeking a thermodynamic rationale^36^. CALPHAD has been extensively utilised in literature for phase prediction and rapid screening of potential alloys by estimating their compositional and microstructural properties which are validated experimentally^36,42^. However, no study can be seen in the literature with a focus on mechanical property prediction solely from the CALPHAD method. This is because predicting mechanical properties is not as straightforward as phase prediction. Phase prediction relies solely on the Gibbs free energy for lower-order compositions. For more complex or higher-order compositions, phases are predicted by extrapolating Gibbs free energy from the lower-order systems^43^. Unlike phase prediction, mechanical property prediction requires rigorous research into the manufacturing (processing) routes, processing parameters, post-processing treatments, testing parameters and extensive knowledge or expertise in the field. Thermo-Calc 2024a offers a property model calculator, which allows the prediction of yield strength and hardness of a composition based on the phases present at a particular temperature^44^.

**Fig. 2:**
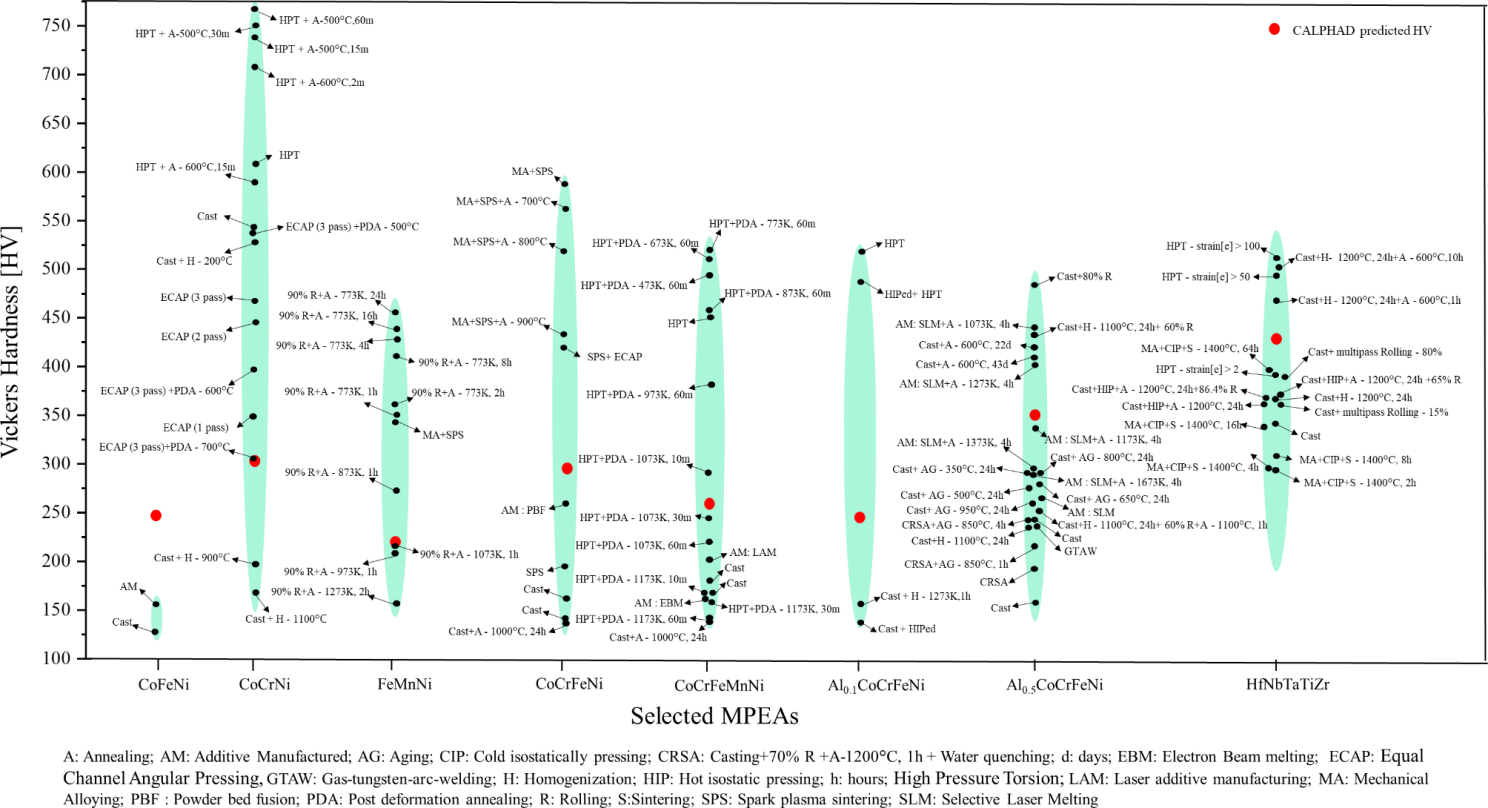
Vickers hardness comparison for selective MPEAs based on the experimental results obtained from various manufacturing methods (black dots) vs. CALPHAD predicted values (in red dots).

Currently, Thermo-Calc does not account for factors such as processing history, parameters, time and other crucial variables for accurately predicting mechanical properties under specific experimental conditions. However, its hardness prediction tool still provides a solid foundation for making informed estimations. To assess the discrepancy between experimentally obtained mechanical properties and Thermo-Calc predictions, we extracted the Vickers hardness values of various alloys, including medium- and high-entropy alloys, from experimental literature across different processing methods (casting, additive manufacturing, powder metallurgy, rolling, and severe plastic deformation techniques such as High-Pressure Torsion (HPT) and Equal Channel Angular Pressing (ECAP)). These were then compared to Thermo-Calc predictions, as shown in Fig. 2. A detailed comparison is provided in tabular form in supplementary Table 1s. Thermo-Calc predictions were seen to be insensitive to the strain rate applied during the manufacturing process which can lead to different hardness values based on the manufacturing process. For a few compositions, the CALPHAD hardness value matched the experimental values, however, it is difficult to generalize which experiments led to the values that are closest to the CALPHAD predictions. For instance, the hardness values obtained from CALPHAD for CoCrNi match with the ECAP processed (for 3 passes) and post-deformation annealed (at 700 °C) samples. As for the FeMnNi medium entropy alloy, its CALPHAD value was closest to the alloy processed via rolling (90% rolled) and then annealed at 1073 K for 1 h (see Fig. 2). Thus, generalizing which experimental processing route leads to hardness values that closely match those predicted by CALPHAD is arduous.

### Data collection using Thermo-Calc 2024a

Despite cobalt’s inclusion in the 1st tier CRM category, its production is not a concern. The Democratic Republic of Congo (DRC) is the world’s largest producer of Co, with its production projected to increase from 11,000 MT in the year 2000 to 98,000 MT by 2020^45^. However, the market of Co presents a considerable risk due to supply-chain complexities. China, which has limited domestic cobalt production has significantly increased its imports from the Democratic Republic of the Congo (DRC) and controls cobalt processing in the region through various Chinese firms. This initiative by China was aimed at securing a competitive advantage in regulating the electric vehicle market.

A potential remedy here would be to recover Co from waste batteries. Suriyanarayanan et al.^24^ recently introduced an innovative and efficient approach for Co extraction with an extraction efficiency > 97%, using a nonionic deep eutectic solvent (ni-DES) comprised of N-methylurea and acetamide. Zhang et al.^25^ developed a supercritical fluid extraction process using supercritical CO_2_ solvent with tributyl phosphate–nitric acid and hydrogen peroxide adduct to recover Li, Co, Mn and Ni with a 90% extraction efficiency. Moreover, Yang et al.^26^ in 2024 estimated the sales volume of new energy passenger vehicles (NEPV) from 2023 to 2035 based on the historical NEPV sales data from 2013 to 2022. Utilizing Weibull distribution to analyze different sales scenarios, they estimated the potential of recycling Co for maintaining a balance between supply and demand. Their analysis predicted the peak potential of recycling of Co to be about 0.167 MT with an economic value ranging from 49.01 billion to 94.60 billion RMB in 2035. Consequently, they concluded that recycling Co is necessary to alleviate the supply risk pressure and take Co off of the CRM list.

Similarly, Petranikova et al.^23^ summarized the efforts in the recovery of Vanadium by selecting more sustainable technologies with lower generation of harmful by-products. They highlighted the importance of combining hydrometallurgical and pyrometallurgical approaches to increase the material recovery rates. With ongoing strategic advancements in recovery and recycling, Cobalt (Co) and Vanadium (V) are expected to transition from their current status as CRMs to non-CRMs. As these management methods evolve, the associated risks related to these materials are anticipated to gradually decrease.

In this context, a large dataset of Vickers hardness values for unary and binary element-based compositions using the property model calculator in Thermo-Calc 2024a (Version 2024.1.132110-55) was compiled by focusing on elements Al, Cr, Cu, Co, Fe, Ni, Ti, V, Mo, Mn, Sn, Zn and Zr.

While extracting the data, it was observed that the property predictor module relies on certain assumptions. Those assumptions are: (i) the material is homogeneous i.e., with no imperfections or defects (ii) it considers only local equilibrium and neglects long-range diffusion. Therefore, to calculate the mechanical properties, it uses simplified theoretical models and databases (containing thermodynamic and kinetic data), which do not capture the complexities related to processing (as-cast, heat-treated, homogenized, or severely deformed samples) and testing conditions (the amount of load and time required in hardness testing). Thermo-Calc predictions can therefore be expected to carry a certain percentage of error when compared to the experimentally synthesized specimen based on its processing history.

For determining the hardness value of a particular composition using Thermo-Calc, the phases present in the system were first identified using their corresponding phase diagram observed at a range of temperatures, computed using an equilibrium calculator. Based on the available features within the software, by considering the system size of 1 mol at a temperature of 300 K and 1 bar pressure, the hardness value was estimated. In some cases, the identified phases in the phase diagram could not be marked while calculating Vickers hardness due to their unavailability in the property model calculator, highlighting one of the several limitations of Thermo-Calc 2024a that needs to be improved.

Consequently, a total of 3,608 instances of different compositions were extracted. This database contains only compositional information and the hardness value of each instance. The highest hardness value obtained was in the range of 400–405 HV. Some of the interesting compositions with higher Vickers hardness values in the database were Cr_42_Ti_58_, Ti_74_Zn_26_, Ni_24_Ti_76_, Cu_16_Ti_84_ with a hardness value of 405HV, 404 HV, 403 HV and 402 HV respectively. The complete dataset is provided as supplementary data. It’s worth noting that no experimental data was considered in this database to avoid mixing synthetic data into the prediction model.

### Data sorting

The database was first checked to avoid repetition. The screened dataset was divided into 80:20 (2,886 and 722 instances) ratio for training, evaluation and verification.

### Tree-based ML model evaluation

Various tree-based regression algorithms such as Decision Tree Regressor (DTR), Random Forest Regressor (RFR), AdaBoost Regressor (ABR), Gradient Boost Regressor (GBR), XGBoost Regressor (XGBR) and Extra Tree Regressor (ETR) were employed. A detailed description of each algorithm with its flowchart is provided in Section “Methodology” as Supplementary information.

The performance of a machine learning (ML) model is influenced by hyperparameters, which are adjustable settings that govern various aspects of the model’s learning process, such as complexity, regularisation, and convergence. Examples of hyperparameters include the maximum depth of trees in decision tree models, the number of trees in ensemble models like Random Forest and Gradient Boost, as well as the number of hidden layers in neural networks and the penalty term used in support vector machines. Proper tuning of these hyperparameters is essential for achieving an optimal balance between model accuracy and generalisation.

To optimize the hyperparameters of the selected algorithms, Random Search CV was employed. Unlike Grid Search, which systematically evaluates all possible combinations of hyperparameter values, Random Search, samples a fixed number of hyperparameter combinations from a defined distribution. This approach significantly reduces computational costs while still allowing the exploration of a diverse range of values, making it particularly advantageous for complex models with many hyperparameters.

**Fig. 3:**
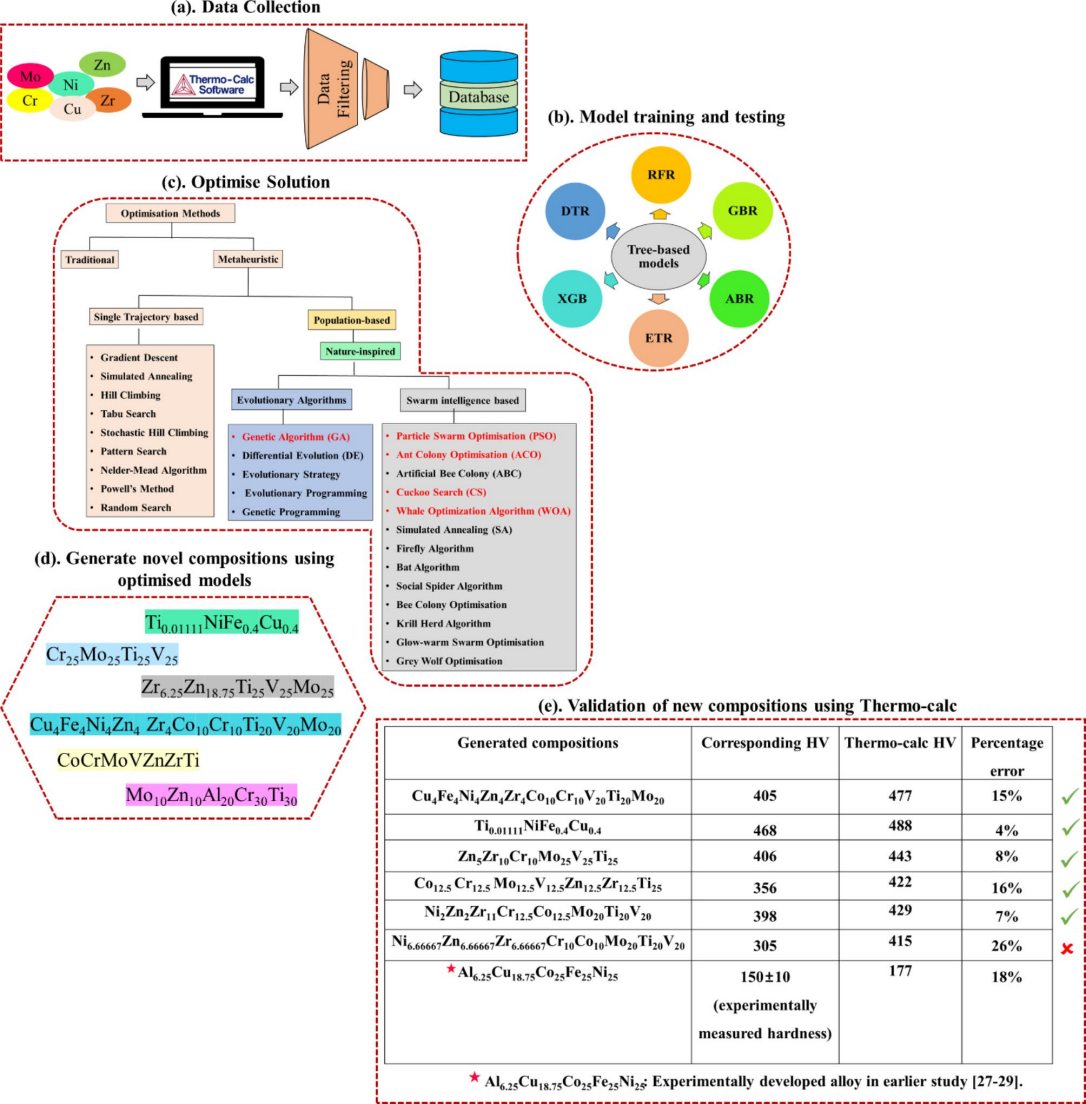
Workflow of the current study: (**a**). data collection and processing; (**b**). training, testing and evaluation of tree-based models; (**c**). classification of optimization models (full-length image is provided in Section “Optimisation techniques”) and selection of best optimization model (models in red font were selected for this study) for obtaining our objective; (**d**). generated novel compositions; (**e**). comparison of the predicted hardness with Thermo-Calc ones for validation; hardness of novel HEA (Al_6.25_Cu_18.75_Co_25_Fe_25_Ni_25_) prepared in the earlier study was measured and compared with Thermo-Calc one for reinforcing the present study.

Each hyperparameter configuration was evaluated using a 5-fold cross-validation approach, where the dataset was divided into five subsets. Each subset was used once as a validation set while the others served as the training set, ensuring a thorough and unbiased assessment of the model’s generalization capabilities. The coefficient of determination (R² score), mean absolute error (MAE), root mean squared error (RMSE), and mean absolute percentage error (MAPE), as detailed in Eq. 1 to 4, were used as performance indicators to quantify the difference between predicted values and observed outcomes. The optimized hyperparameters for each regressor model are listed in Table 1. This methodology helped in identifying the best-performing hyperparameter settings, ensuring that the model would perform consistently across different data splits. The working principle of this investigation is illustrated in Fig. 3.

1 $${\text{R}}^{2} \_{\text{Score}} = \frac{{\sum {\left( {y_{i} - \hat{y}_{i} } \right)^{2} } }}{{\sum {\left( {y_{i} - \mu } \right)^{2} } }}$$

2 $${\text{MAPE = }} = \frac{1}{n}\sum\nolimits_{{i = 1}}^{n} {\frac{{\left| {\left( {y_{i} - \hat{y}_{i} } \right)} \right|}}{{\left( {y_{i} } \right)}}}$$

3 $${\text{MAE}} = \frac{1}{n}\sum\nolimits_{{i = 1}}^{n} {|y_{i} - \hat{y}_{i} |}$$

4 $${\text{RMSE}} = \sqrt {\frac{1}{n}\sum\nolimits_{{i = 1}}^{n} {\left( {y_{i} - \hat{y}_{i} } \right)^{2} } }$$

**Table 1:** Summary of the optimized hyperparameters for the chosen ML algorithms.

Regression algorithm	Hyperparameters
Decision Tree Regressor (DTR)	min_samples_split: 4; min_sample_leaf: 3; max_features: None
Random Forest regressor (RFR)	n_estimators: 651; min_samples_split: 2; min_sample_leaf: 1; max_depth: 70; bootstrap: True max_features: ‘sqrt’
Gradient Boost Regressor (GBR)	Subsample: 0.97; n_estimators: 609; min_samples_split: 9; min_sample_leaf: 3; max_depth: 8; learning_rate: 0.042 max_features: ‘log2’
AdaBoost Regressor (ABR)	learning_rate: 0.78; loss: linear; n_estimators: 280
XGBoost (XGB)	colsample_bytree: 0.87; gamma: 4.7; learning_rate: 0.22; max_depth: 7; min_child_weight: 5; n_estimators: 1738; subsample: 0.94
Extra Trees Regressor (ETR)	n_estimators: 1301; min_samples_split: 4; min_sample_leaf: 1; max_depth: None; bootstrap: False max_features: ‘log2’

**Fig. 4:**
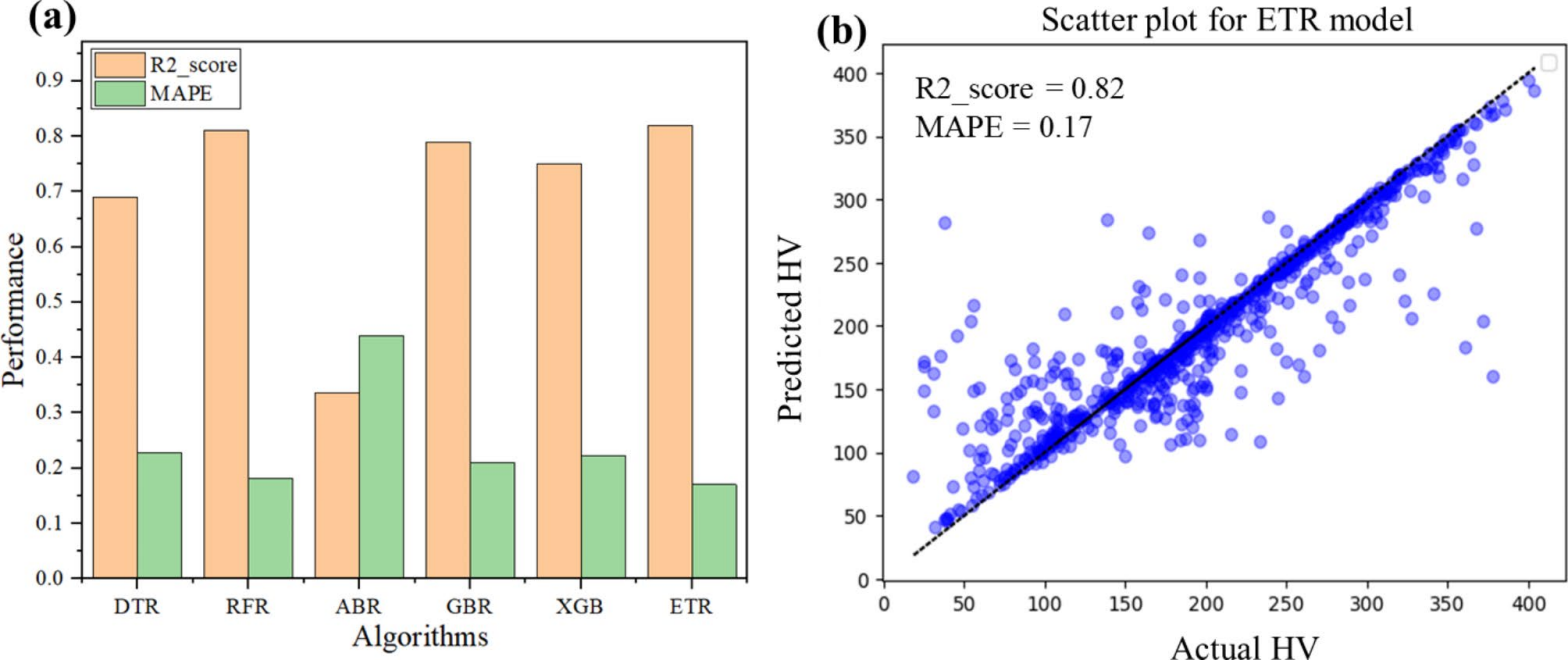
(**a**) Performance of various tree-based ML models: R2_score (in light orange), MAPE (in green), (**b**) actual vs. predicted hardness of test data for the ETR model.

The results obtained from various algorithms are presented in Fig. 4a. The Extra Trees Regressor (ETR) demonstrated a superior performance and achieved an R² score of 0.82 and Mean Absolute Percentage Error (MAPE) of 0.17, utilizing optimized hyperparameters determined through Random Search Cross-Validation (see the scatter plot in Fig. 4b). It is important to note that while several studies report even higher R² scores, those models typically incorporate numerous descriptors, such as atomic size difference (δ), electronegativity difference (∆χ), valence electron concentration (VEC), mixing enthalpy (∆Hmix), mixing entropy (∆Smix), melting temperature (∆Tm), Young’s modulus (E), shear modulus (G), differences in shear modulus (δG), lattice distortion energy (µ), the Peierls-Nabarro factor (F), and other parameters (Ω-parameter, ϕ-parameter, and γ-parameter), which together enhance performance metrics. In contrast, our approach relies solely on compositional information and hardness values, achieving an R² score of 0.82. This underscores the model’s robustness to predict hardness values without requiring any additional descriptors.

To explore novel multi-component compositions, we employed a metaheuristic optimization strategy aimed at generating alloys with superior hardness compared to those in the training and testing datasets, which consisted exclusively of unary and binary compositions. The optimization techniques that facilitate the exploration of more complex and high-performance MPEAs are detailed in the subsequent sections. The hardness values of the newly optimized MPEAs were then compared against CALPHAD-based predictions, and these findings are discussed comprehensively in Section “Results and discussions”.

### Optimisation techniques

The classification of various optimisation models followed in this work is shown in Fig. 3c with red fonts highlighting the algorithms used in this investigation. Traditional optimisation techniques, which include general methods and non-general or specified methods tailored for specific types of problems, have certain limitations such as the requirement of the objective function to be differentiable and lack of ability to obtain a globally optimum solution. Some of the popular traditional optimisation techniques such as Newton Raphson, Successive Quadratic Programming algorithm, Steepest Descent Algorithm, Stochastic Newton optimisation method and Sequential Unconstrained Minimization technique^46,47^ are well known. Recently, some non-traditional methods of optimisation popularly known as meta-heuristic optimisation techniques have gained increasing popularity in solving complex problems. The term metaheuristic combines meta and heuristic, both originated from Greek. Meta symbolizes higher or beyond, and heuristic signifies intelligent guesswork based on past experience or intuitive solution of a problem. Therefore, metaheuristic optimisation can be considered as something beyond intuitive, combined with certain mathematical rules or higher-level frameworks. It can broadly be classified into two categories: single trajectory-based and population-based optimisation. Single-trajectory based optimisation (such as Hill Climbing, Gradient Descent, Tabu Search, Random Search etc.) starts with a single solution at each iteration, and the current solution is replaced by another best solution found in the neighbourhood for that iteration. Contrarily, population-based optimisation techniques are inspired by natural-selection and biological evolution, where a set of solutions are randomly initialized and updated through an iterative process. Genetic algorithm, Differential evolution and others belong to evolutionary optimisation techniques, while Particle swarm optimisation, Ant colony optimisation, Cuckoo search and others are examples of swarm-intelligence-based optimisation techniques^47,48^. These techniques are exploration-and-exploitation oriented, which introduces diversification in the search space, resulting in the attainment of global optimum solutions by avoiding local optimum solutions for complex real-world problem^49^.

**Fig. 5:**
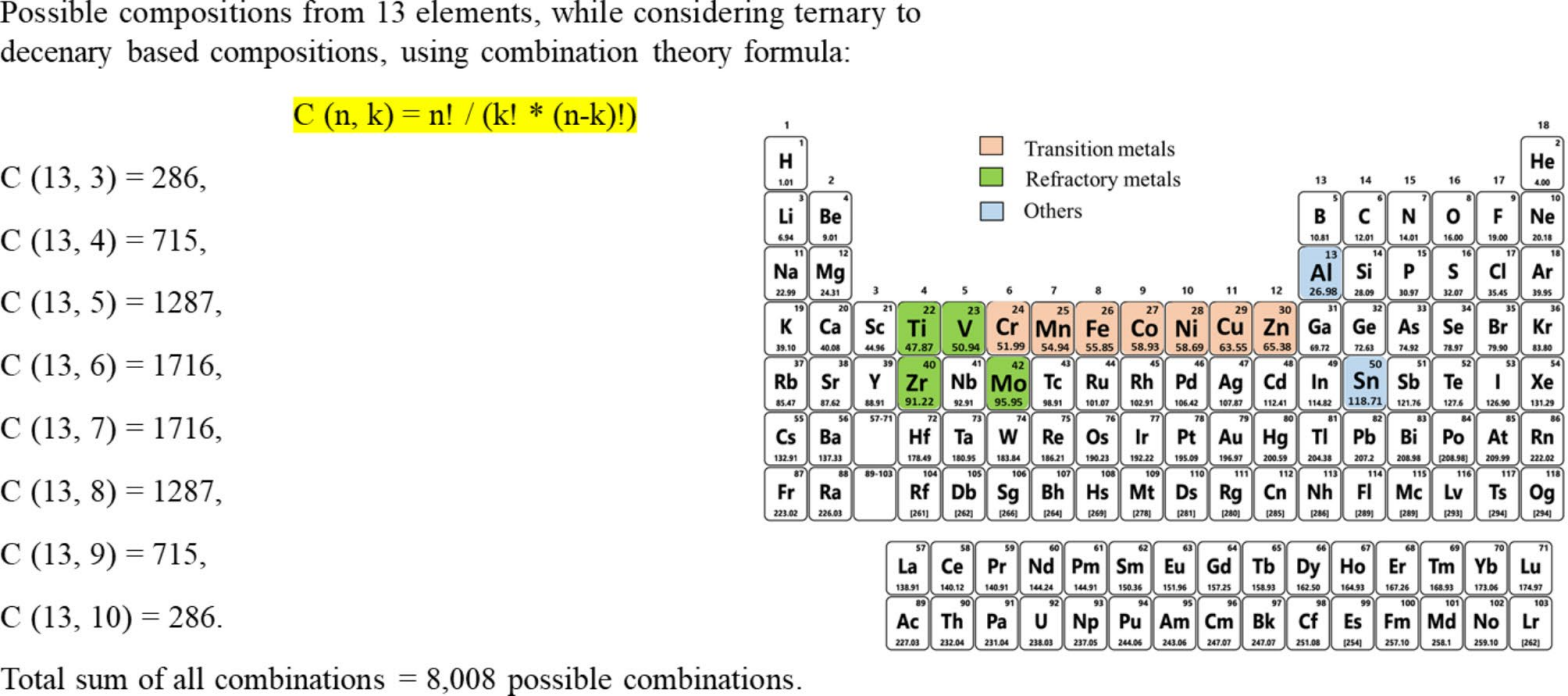
Total possible compositions from selected 13 elements.

**Fig. 6:**
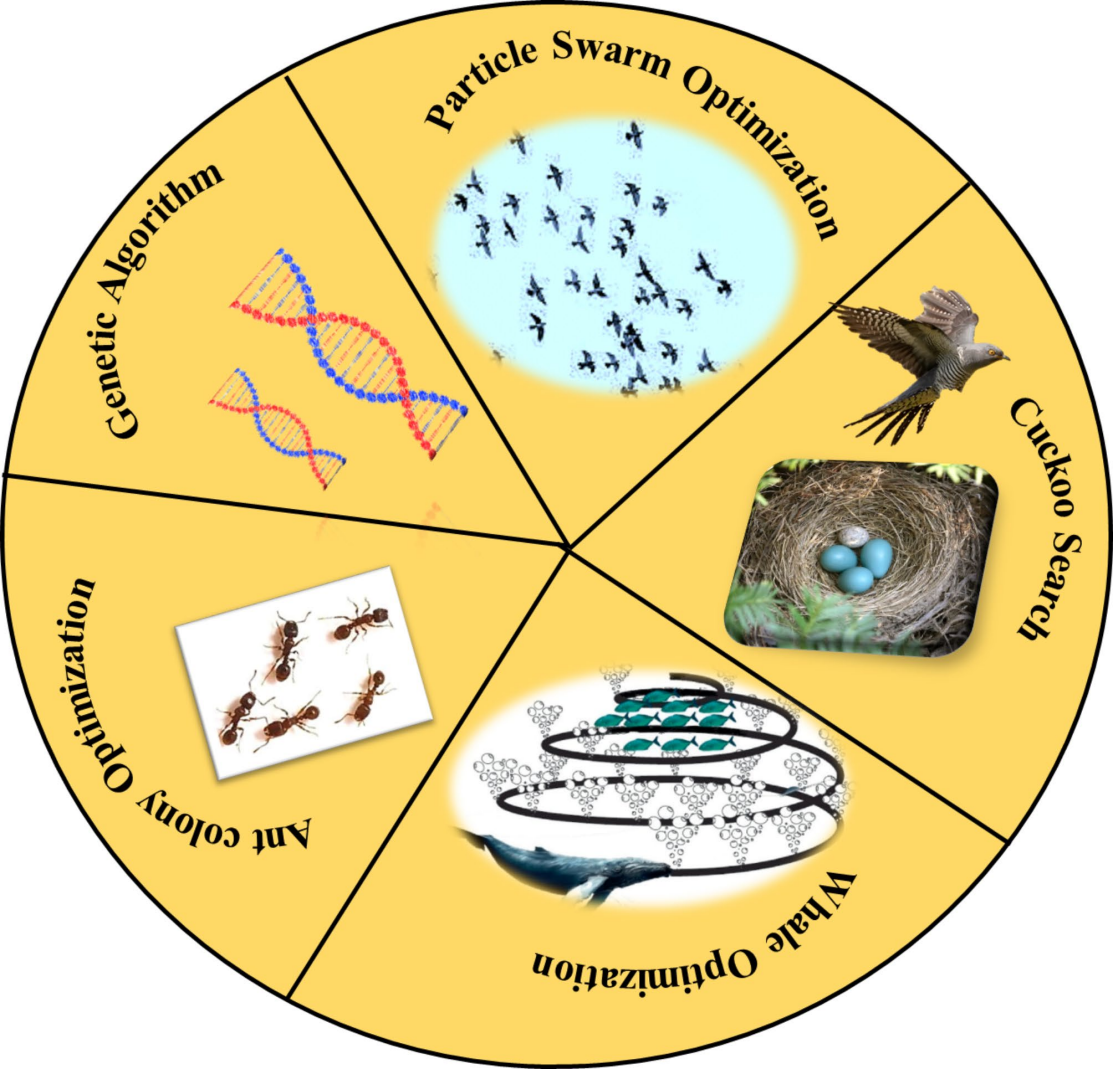
Various metaheuristic optimisation models applied in this work: (1). Genetic Algorithm (GA), (2). Particle Swarm Optimisation (PSO), (3). Cuckoo Search Optimisation (CSO), (4). Whale Optimisation Algorithm (WOA) (5). Ant Colony Optimisation (ACO).

Among all available various nature-inspired metaheuristic optimisation techniques, genetic algorithms, particle swarm optimisation, Cuckoo search and a few others have been proven to successfully solve a wide range of complex real-world problems. However, some of the recently introduced metaheuristic optimisation techniques such as Ant colony optimisation, Artificial Bee Colony optimisation, Spotted Hyena optimisation, whale optimisation need substantiation for their convergence. Rao^50^ in his book chapter has highlighted that the fundamental idea of these methods is the same while naming them differently. In this work, we compared which optimisation technique works best for our ETR model to discover new multicomponent compositions from the unexplored compositional space of nearly 8,008 types (see Fig. 5) as per combination theory while only ternary to decenary-based compositions were considered for 13 elements. Figure 6 describes five metaheuristic optimization techniques used in this study. A detailed description of each optimization technique with its working principle is elaborated in section 3s of the supplementary information.

## Results and discussions

### Evaluation of several reduced-CRM-MPEAs

Optimisation function was set up to obtain novel multi-principal element alloys with Vickers hardness > 400 HV, by enforcing composition constraint such that the sum of 3, 4, 5, 6, 7, 8, 9, or 10 elements become 100, while keeping the proportion of each element equal or near equal to generate multicomponent compositions.

Among all the optimisation models, cuckoo search optimisation (CSO) provided predictions near the Thermo-Calc predictions. Recent literature suggests that the cuckoo search optimisation (CSO) performs better than PSO, GA, ACO, ABC and WOA^51–54^. Gandomi et al.^51^ provided an extensive comparison and concluded that CSO performs better than GA and PSO, as GA requires a higher number of iterations and its implementation is computationally expensive^53^. On the other hand, PSO requires less computational effort but considerable execution time to find a solution from a large space for a complex optimisation problem. Civicioglu and Desdo^8^ suggested that CSO provides more robust results than PSO and ABC. Bhargava et al.^55^ showed that CSO offers a reliable method for solving thermodynamic calculations for complex phase equilibrium applications.

The ternary to decenary multicomponent compositions generated by the various algorithms, including GA, PSO, WOA, ACO and CSO, are presented in Tables 2, 3, 4, 5 and 6, respectively. These compositions were subsequently validated using the property model calculator in Thermo-Calc (TC) software. By comparing the hardness values of the newly generated compositions to those predicted by TC, it is evident that the CSO algorithm produced the most reliable multicomponent compositions, with a prediction error of less than ± 20%. Furthermore, compositions generated by CSO exhibited superior hardness compared to those derived from other optimization methods.

For instance, CSO successfully generated compositions such as Cu_4_Fe_4_Ni_4_Zn_4_Zr_4_Co_10_Cr_10_V_20_Ti_20_Mo_20_ (10 elements), Zn_5_Cr_10_Zr_10_Mo_25_Ti_25_V_25_ (6 elements), Zr_6.25_Zn_18.75_Ti_25_V_25_Mo_25_ (5 elements), Ti_0.01111_NiFe_0.4_Cu_0.4_ (4 elements), which achieved hardness values of 477 HV, 443 HV, 434 HV and 488 HV respectively. In contrast, while GA, PSO, and WOA demonstrated consistency in their predictions for several compositions, they often failed to accurately predict compositions with higher hardness values. Notably, these algorithms became increasingly erroneous with the inclusion of more elements in the alloy design.

ACO method proved to be reliable for ternary and quaternary compositions, yielding relatively low prediction errors. However, as the complexity escalated from quinary to decenary compositions, the percentage error substantially increased, and ACO was unable to generate viable compositions with more than seven elements.

**Fig. 7:**
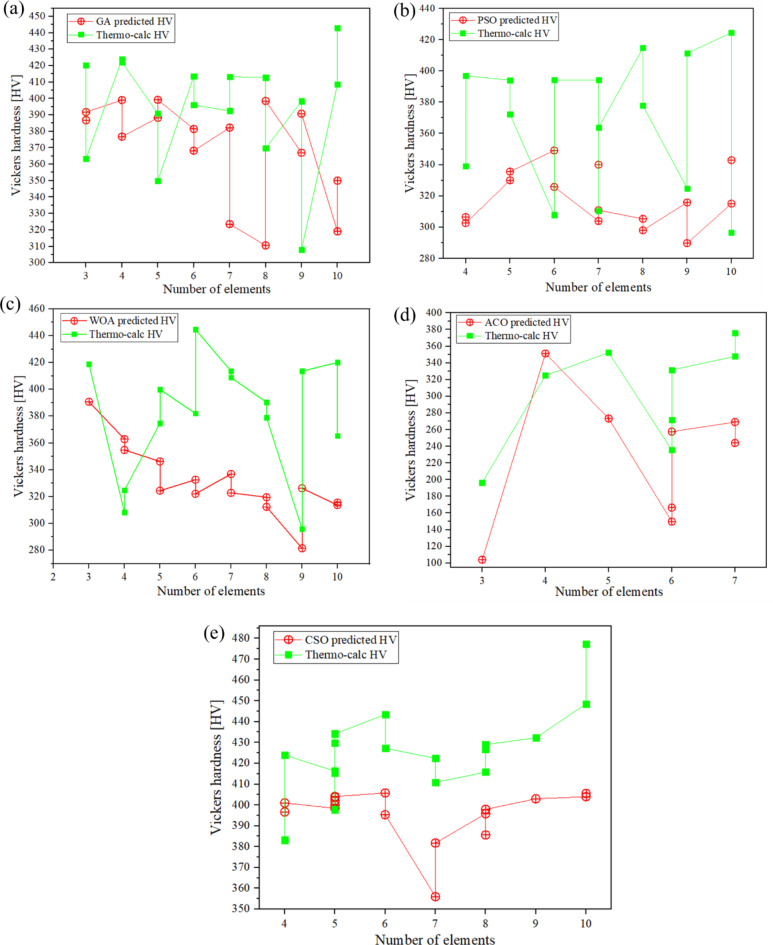
Comparison of hardness value for newly generated MPEA compositions compared for ML and Thermo-Calc predictions using various techniques: (**a**) Genetic algorithm (GA), (**b**) Particle swarm optimisation (PSO), (**c**) Whale optimization (WOA), (**d**) Ant colony optimization (ACO), (**e**) Cuckoo search optimization (CSO).

As a result, CSO emerged as the most reliable algorithm among those tested, capable of generating multicomponent compositions with reduced CRM content and enhanced hardness values. Figure 7 illustrates the hardness values generated by each algorithm in comparison to those evaluated by Thermo-Calc for ternary to decenary compositions, providing a comprehensive overview of algorithm performance across varying levels of compositional complexity.

**Table 2:** Compositions generated using GA optimisation.

No. of elements	Composition suggested from GA	Corresponding HV	Thermo-calc HV	Percentage error (%)
3	Mn_23.41682_Sn_23.41682_Ti_53.16636_	387	421	8%
3	Cu_9.10794325_Mo_9.10794325_Ti_81.7841135_	392	364	8%
4	Cu_6.11253929_Ni_6.11253929_Cr_32.1722748_Ti_55.60264661_	399	425	6%
4	Cr_11.11111_Co_22.22222_Ti_22.22222_Mo_22.22222_V_22.22222_	377	423	11%
5	Ni_5.79651873_Zn_5.79651873_Mo_5.79651873_Mn_30.09710402_Ti_53.2668911_	388	391	0.8%
5	Mo_20_Mn_20_Ti_20_Sn_20_V_20_	399	350	14%
6	Ti_6.66667_Cr_16.66667_Co_16.66667_Mo_20_V_20_Zr_20_	381	414	8%
6	Cr_10_Zr_10_Mo_20_Ti_20_Sn_20_V_20_	368	397	7%
7	Cu_5_Zr_5_Co_15_Cr_15_Ti_20_Mo_20_V_20_	382	393	3%
7	Zn_5_Zr_5_Co_10_Ti_20_Cr_20_Mo_20_V_20_	324	414	22%
8	Ni_5_Zr_5_Cr_10_Co_10_Zn_10_Ti_20_V_20_Mo_20_	310	413	25%
8	Co_10_Sn_10_V_10_Zn_10_Zr_10_Cr_12.5_Ti_17.5_Mo_20_	398	370	8%
9	Cu_5_Ni_5_Zn_5_Zr_5_Co_12_Cr_12_Mo_13_V_13_Ti_30_	367	398	8%
9	Cu_5_Co_5_Ni_5_Zr_5_Cr_8_Mo_10_Ti_10_V_10_Sn_42_	391	308	27%
10	Mn_2_Sn_2_Zr_4_Cu_6_Zn_6_Cr_10_Co_10_Ti_20_V_20_Mo_20_	319	409	22%
10	Cu_3_Ni_3_Fe_3_Zn_3_Zr_3_Co_5_Cr_5_Mo_25_Ti_25_V_25_	350	444	21%

**Table 3:** Compositions generated using PSO optimisation.

No. of elements	Composition suggested from PSO	HV predicted by PSO	Thermo-calc HV	Percentage error (%)
4	Mo_5.55556_Zn_5.55556_Mn_44.44444_V_44.44444_	306	339	10
4	Fe_6.25_Ni_6.25_Cr_43.75_ Zn_43.75_	303	397	24
5	Ni_5.88235_Zr_5.88235_Mn_5.88235_Cr_41.17647_Ti_41.17647_	330	394	16
5	Cr_4.76190_Ni_4.76190_Co_4.76190_Mn_42.85714_Ti_42.85714_	335	372	10
6	Cu_4.54545455_Mn_4.54545455_Sn_4.54545455_Fe_4.54545455_ Cr_40.90909091_Ti_40.90909091_	349	308	13
6	V_4.16667_Ni_4.16667_Fe_4.16667_Mo_4.16667_Cr_41.66667_ Ti_41.66667_	326	395	17
7	Cu_8_Zr_8_Co_12_Cr_12_Ti_20_Mo_20_V_20_	304	394	23
7	Co_4_Cu_4_Fe_4_Mn_4_Ni_4_Cr_40_Ti_40_	340	310	10
7	Co_5_V_5_Zn_5_Zr_5_Cr_10_Mo_20_Ti_50_	311	364	14
8	Ni_6.66667_Zn_6.66667_Zr_6.66667_Cr_10_Co_10_Mo_20_ Ti_20_V_20_	305	415	26
8	Cu_5_Zn_5_Zr_5_Co_12_Cr_12_Mo_13_V_13_Ti_35_	298	378	21
9	Cu_5_Ni_5_Co_5_Zn_5_Ti_10_V_10_Cr_10_Mo_10_Sn_40_	316	325	3
9	Cu_5_Ni_5_Zn_5_Zr_5_Co_10_Cr_10_Mo_15_V_15_Ti_30_	290	411	29
10	Al_4.16666667_Cu_4.16666667_Cr_4.16666667_Fe_4.16666667_ Mn_4.16666667_Ti_4.16666667_Zn_4.16666667_Zr_4.16666667_ Mo_33.33333333_V_33.33333333_	315	424	26
10	Co_10_Cr_10_Cu_10_Fe_10_Ni_10_Mo_10_Ti_10_V_10_Zr_10_Zn_10_	343	296	16

**Table 4:** Compositions generated using WOA optimisation.

No. of elements	Composition suggested from WOA	HV predicted by WOA	Thermo-Calc HV	Percentage error (%)
3	Cu_11.2_Mn_36.8_Ti_52_	391	419	7
4	Mo_5.92105_Al_13.81579_Co_17.10526_Ti_63.15789_	363	192	89
4	Fe_10_Zn_20_Cr_30_Ti_40_	355	325	9
5	Ni_3.41880_Mn_4.27350_Zn_10.25641_V_29.05983_ Mo_52.99145_	346	375	8
5	Cr_9.09091_Mn_9.09091_Zr_9.09091_Mo_36.36364_V_36.36364_	324	400	19
6	Fe_5.55556_Mn_5.55556_V_5.55556_Zn_5.55556_ Ti_38.88889_Cr_38.88889_	333	382	13
6	Cr_7.14286_Mn_7.14286_Ti_7.14286_Zr_7.14286_Mo_35.71429_V_35.71429_	322	445	28
7	Co_4.34783_Cr_4.34783_Ni_4.34783_Ti_4.34783_Zn_4.34783_Mo_39.13043_ V_39.13043_	337	414	18
7	Co_5_Cr_10_V_10_Zn_10_Zr_10_Mo_20_Ti_35_	323	409	21
8	Al_5_Co_5_Cu_5_Fe_5_Mn_5_Ni_5_Mo_35_V_35_	319	390	18
8	Al_12.5_Co_12.5_Cr_12.5_Mo_12.5_Ti_12.5_V_12.5_Zn_12.5_Zr_12.5_	312	379	18
9	Fe_4.34783_Ni_4.34783_Mo_4.34783_Sn_4.34783_Ti_4.34783_Zn_4.34783_ Zr_4.34783_Cu_34.78261_V_34.78261_	281	296	5
9	Cu_5_Ni_5_Zn_5_Zr_5_Co_6_Cr_6_Mo_9_V_9_Ti_50_	326	413	21
10	Al_3.57143_Co_3.57143_Cr_3.57143_Cu_3.57143_Fe_3.57143_Ni_3.57143_ Ti_3.57143_Zr_3.57143_Mo_35.71429_V_35.71429_	314	420	25
10	Al_5_Co_5_Cr_5_Fe_5_Mn_5_Ti_5_Zr_5_Zn_5_Mo_30_V_30_	315	365	14

**Table 5:** Compositions generated using ACO optimisation.

No. of elements	Composition suggested from ACO	HV predicted by ACO	Thermo-calc HV	Percentage error (%)
3	Co_13_Al_20_Cu_67_	168	196	14
4	Fe_10_Zn_20_Cr_30_Ti_40_	278	325	14
5	Fe_4_Co_15_V_24_Sn_28_Cr_29_	273	352	22
6	Sn_4_Mn_7_Ti_13_Ni_21_Al_26_Zr_29_	150	235	36
6	Cr_8_Cu_8_Mn_9_Sn_16_Al_18_Zn_41_	167	272	39
6	Cu_6_Mo_6_Ni_6_Zr_20_Sn_30_Cr_32_	258	332	22
7	Fe_3_Mo_4_Mn_8_Cu_9_Zn_19_Cr_25_Co_32_	269	348	23
7	Ti_2_V_6_Fe_14_Mn_15_Cr_17_Zr_18_Mo_28_	244	376	35

**Table 6:** Compositions generated using CSO optimisation.

No. of elements	Composition suggested from CSO	HV predicted by CSO	Thermo-calc HV	Percentage Error (%)
4	Mo_25_Sn_25_Ti_25_V_25_	397	383	4
4	Cr_25_Mo_25_Ti_25_V_25_	401	424	5
4	Ti_0.01111_NiFe_0.4_Cu_0.4_	**468**	**488**	**4**
5	Co_12.5_Cr_12.5_Mo_25_Ti_25_V_25_	398	416	4
5	Mo_10_Zn_10_Al_20_Cr_30_Ti_30_	400	398	0.5
5	Co_11.11111_Cr_11.11111_ Mo_22.22222_V_22.22222_Ti_33.33333_	404	430	6
5	Zn_6.25_Cr_18.75_Mo_25_Ti_25_V_25 (similar to novel one)_	402	415	3
5	Zr_6.25_Zn_18.75_Ti_25_V_25_Mo_25 (similar to novel one_	404	434	7
6	Zn_5_Cr_10_Zr_10_Mo_25_Ti_25_V_25_	406	443	8
6	Co_16.66667_Cr_16.66667_Mo_16.66667_Ti_16.66667_V_16.66667_ Zr_16.66667_	395	427	7
7	CoCrMoVZnZrTi	356	422	16
7	Cu_5_Zr_5_Co_12_Cr_12_Mo_13_V_13_Ti_40_	382	411	7
8	Co_10_Mn_10_V_10_Zn_10_Zr_10_Cr_12.5_Ti_17.5_Mo_20_	396	416	5
8	Co_12.5_Cu_12.5_Cr_12.5_Mo_12.5_Ti_12.5_V_12.5_Zn_12.5_Zr_12.5_	385	427	10
8	Ni_2_Zn_2_Zr_11_Cr_12.5_Co_12.5_Mo_20_Ti_20_V_20_	398	429	7
9	Cu_5_Ni_5_Zn_5_ Zr_5_Co_8_Cr_8_Mo_10_V_10_Ti_44_	403	432	7
10	Co_3.57143_Cr_3.57143_Cu_3.57143_Fe_3.57143_Ni_3.57143_ Zn_3.57143_Zr_3.57143_Mo_25_Ti_25_V_25_ OR (CoCrCuFeNiZnZr)_0.1428572_MoTiV	404	448	10
10	Cu_4_Fe_4_Ni_4_Zn_4_ Zr_4_Co_10_Cr_10_Ti_20_V_20_Mo_20_	405	477	15

Significant values are in bold.

**Fig. 8:**
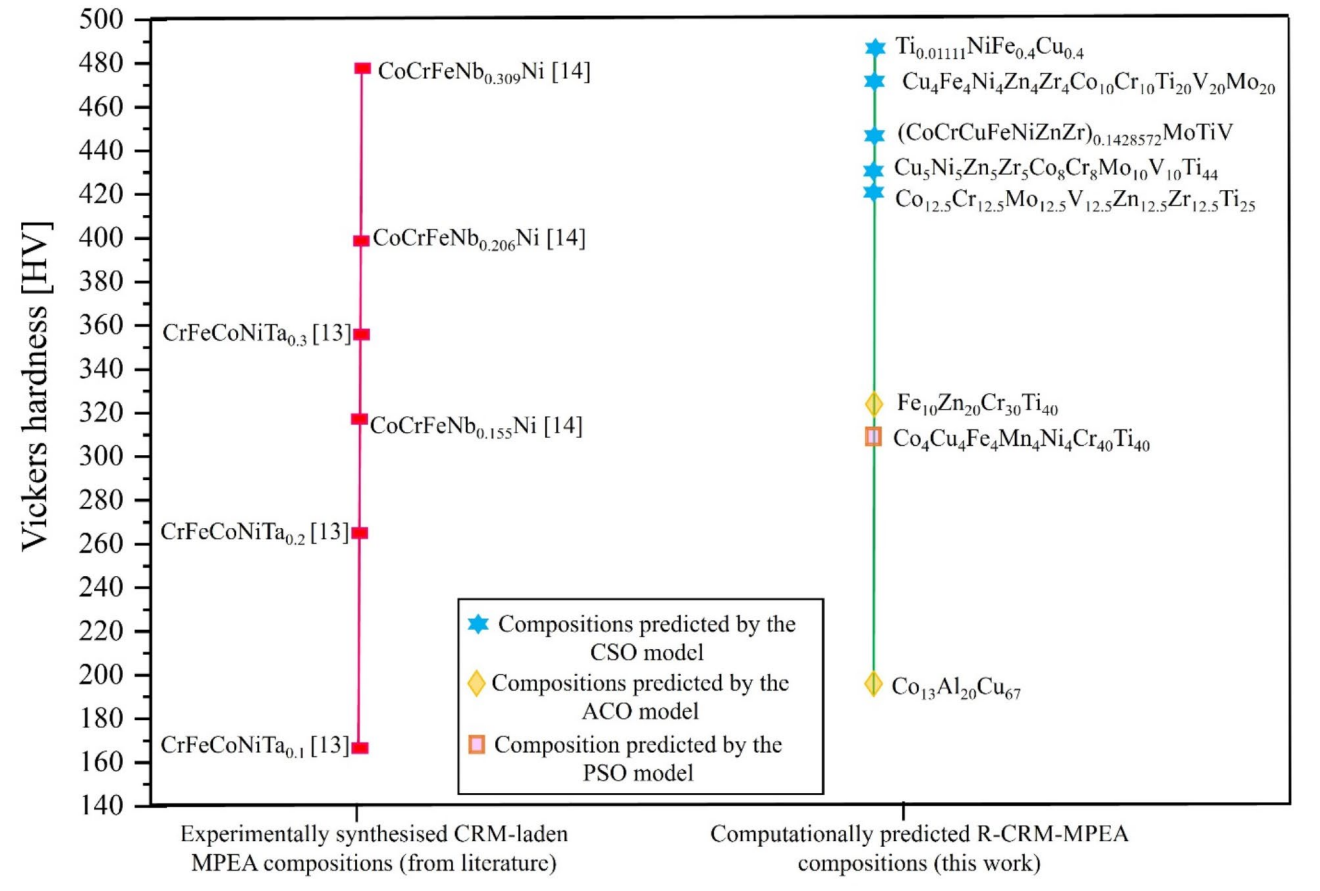
Comparison of R-CRM-MPEA with CRM-laden MPEAs to demonstrate the feasibility of partially substituting CRMs with readily available elements while attaining comparable mechanical properties.

We further compared the hardness values of the newly generated R-CRM-MPEAs with those of CRM-containing MPEAs reported in the literature to assess the feasibility of partially substituting CRMs. Figure 8 provides a comparison of the mechanical properties between experimentally synthesised CRM-laden alloys reported in the literature and the newly predicted R-CRM-MPEAs in this work.

The results indicate that the proposed approach can yield comparable hardness. For example, CoCrFeNb_0.309_Ni containing two 1st-Tier CRMs exhibits a hardness of 480 HV, whereas the newly predicted composition, Ti_0.0111_NiFe_0.4_Cu_0.4_, achieves a superior hardness of 488 HV without including any 1st-Tier CRMs. The computational approach employed using ML in this work has great potential to design R-CRM-MPEA compositions. This approach can be leveraged to eliminate the use of CRMs in diverse applications such as catalysis, semiconductors, transportation and other carbon-intensive sectors.

### Benchmarking of the newly synthesised alloy Al_6.25_Cu_18.75_Fe_25_Co_25_Ni_25_

To test our predictions, a random and a new FCC phase alloy, Al_6.25_Cu_18.75_Fe_25_Co_25_Ni_25_^27–29^ was selected for experimental validation. Vickers hardness testing was performed utilising a Wilson hardness testing apparatus, with a load of 0.1 kgf. The Vickers hardness value was determined by averaging the measurements from nine indents on a polished surface. The experimentally measured Vickers hardness of Al_6.25_Cu_18.75_Fe_25_Co_25_Ni_25_ was subsequently compared with the hardness values evaluated by Thermo-Calc (TC) and our ML model (refer to Table 7). It was observed that both the TC-evaluated and ML-predicted hardness values were in strong agreement with the experimentally measured hardness with an error of less than 20%.

**Table 7:** Comparison of experimentally measured hardness with TC and ML predicted hardness.

Alloy	Experimentally evaluated [HV_0.1_]	Predicted [TC and ML]		Percentage error (%)
Al_6.25_Cu_18.75_Fe_25_Co_25_Ni_25_	Mean	TC- predicted	177 HV	18
	150 ± 10 HV	ML-predicted	179 HV	19

While numerous studies in the literature on MPEAs have used machine learning, a majority of these remain concentrated on developing algorithms for phase classification^56–58^ or predicting mechanical properties, such as hardness, yield strength, or elastic modulus^59–63^. Relatively few investigations have aimed to generate or optimize novel MPEA compositions. Most of these efforts have concentrated on achieving high hardness values which differs from the focus of this investigation where the main objective was to develop substitute alloys to eliminate the use of CRMs and hardness was used as an indicator to demonstrate that comparable properties can still be obtained.

Ren et al.^64^ used a dataset of 205 HEA samples featuring 19 characteristics commonly employed in HEA property prediction. They implemented a tree-based machine learning model to predict hardness and integrated it with Particle Swarm Optimization (PSO) for component optimization. Due to the limited availability of real experimental data, they resorted to synthetic data through random oversampling to improve the performance of their Component Optimization Model (COM), which raises concerns about the reliability of their prediction. Their database primarily included Al, Co, Cr, Cu, Fe and Ni.

Similarly, Wen et al.^65^ focussed on the same Al-Co-Cr-Cu-Fe-Ni HEA family and its subgroups but worked with a limited dataset of only 155 samples. They proposed a property-oriented materials design strategy that combined machine learning with the Design of Experiments (DOE) to discover alloys with high hardness within this HEA system. Their resulting alloy exhibited a hardness approximately 10% higher than the best value found in the original training set.

In contrast, Yang et al.^66^ used a dataset of 370 HEAs, including compositions such as Al-Co-Cr-Cu-Fe-Ni, Al-Co-Cr-Fe-Mn-Ni, and their derivatives, along with vanadium-containing alloys, which are recognised for their high hardness. They applied techniques such as Inverse Projection (IP) and High-Throughput Screening (HTS) and encountered substantial prediction errors of up to 58% due to the risks associated with extrapolation beyond the boundaries of the training dataset and insufficient data diversity.

To effectively navigate the design space for alloys with high hardness, it is essential to enrich the training datasets with refractory alloys, given their inherent high-temperature stability and robust mechanical properties. However, a major impediment to the reliable application of ML in materials science continues to be the scarcity of relevant data, particularly for HEAs/MPEAs. Roy et al.^67^ addressed this issue by employing a generative adversarial network (GAN) to explore an 18-dimensional design space involving Co-Fe-Ni-Si-Al-Cr-Mo-Ti-Nb-V-Zr-Mn-Cu-Sn-Ta-Hf-W-Zn MPEAs using a limited dataset of 241 alloys. They successfully designed two new MPEAs with hardness values exceeding 941 HV.

In contrast to these studies, our research emphasizes sustainable materials design by assessing whether compositions with reduced or no CRMs can achieve competitive hardness values. This objective addresses a critical gap in the current alloy design landscape—the need for environmentally sustainable materials that minimize reliance on CRMs while maintaining desirable mechanical properties. This approach not only advances sustainability goals but also enhances supply chain resilience, representing a significant step forward in alloy innovation.

## Conclusion

This study represents a significant effort to support Net-Zero initiatives by developing new compositions with reduced critical raw materials (CRMs). The research relies on a computational framework that involves sourcing a dataset of Vickers hardness values for unary (pure) and binary material compositions from Thermo-Calc 2024a and the TCHEA7 database. This dataset was used to build machine learning models to identify complex compositions of alloys with reduced-CRM without negating the mechanical properties.

Among all regression models, the Extra Trees Regressor (ETR) demonstrated superior performance, achieving an R² score of 0.82 and a MAPE of 0.17 for the test data. Various metaheuristic optimization techniques were subsequently employed to inversely predict novel multicomponent alloy compositions free of critical raw materials (CRMs) but with hardness comparable to CRM-containing multi-principal element alloys (MPEAs). Of all the optimization models, Cuckoo Search Optimization (CSO) demonstrated a high level of concordance with Thermo-Calc predictions, with an average deviation of ± 20%. A literature-sourced CRM-laden composition, CoCrFeNb_0.309_Ni, containing two 1st -Tier CRMs (Co and Nb), showed a hardness value of 480 HV and a new alloy was generated using a machine learning method namely, Ti_0.01111_NiFe_0.4_Cu_0.4_, with a hardness of 488 HV that showed great opportunity to eliminate CRMs in developing MPEAs.

The validity of this study was reinforced by comparing computational predictions—derived from our machine learning methodology and Thermo-Calc evaluations—with the experimentally measured Vickers hardness of a test alloy Al_6.25_Cu_18.75_Fe_25_Co_25_Ni_25_, which contains a single 1st-Tier CRM, cobalt (Co). Therefore, this investigation offers valuable insights into the potential for designing novel MPEAs with reduced or even no CRMs, significantly contributing to sustainable materials innovation to support Net Zero in the metal sector. Future research will focus on further experimental validation to corroborate the findings for the newly generated compositions.

## Electronic supplementary material

Below is the link to the electronic supplementary material.

Supplementary Material 1

## Data Availability

The database can be accessed at https://gitfront.io/r/user-6296136/13cNmHofQtp3/Thermo-calc-database. Additional details about the capabilities of Thermo-Calc software can be found in the following resources: (1) Thermo-Calc Product Overview - Property Model Calculator, (2) Brochure: Properties that Thermo-Calc can Calculate.

## Abbreviations

ABR: AdaBoost Regressor
ACO: Ant colony optimisation
CCAs: Complex concentrated alloys
CRM: Critical raw materials
CSO: Cuckoo search optimisation
DTR: Decision Tree Regressor
ETR: Extra Tree Regressor (ETR)
GA: Genetic algorithm
GBR: Gradient Boost Regressor
HEAs: High entropy alloys
MPEAs: Multi-principal element alloys
ML: Machine learning
PSO: Particle swarm optimisation
R-CRM-MPEAs: Reduced-CRM multi-principal element alloys
RFR: Random Forest Regressor
TC: Thermo-Calc
WOA: Whale optimization algorithm
XGBR: XGBoost Regressor

## Declarations

### Content of the publication

During the preparation of this work, the author(s) used AI assistance toimprove the readability and language of the manuscript. The authorsreviewed and edited the final content and took full responsibility for thecontent of the published article.
