# Supplementary material for: Critical raw material-free multi-principal alloy design for a net-zero future
Source: Sci Rep. 2025 Jan 24;15:3132. doi: 10.1038/s41598-025-87784-0 (PMC11760344; doi:10.1038/s41598-025-87784-0)
Supplement: Supplementary file 1 — Supplementary Material 1 [file 41598_2025_87784_MOESM1_ESM.docx]

**Supplementary Information**

**Glossary used:**

LENS: Laser engineered net shaping (LENS) additive manufacturing (AM)

DED-AM: Direct energy deposition

PDA: Post deformation annealing

CIP: Cold isostatically pressing

EBM: Electron Beam melting

ECAP: Equal Channel Angular Pressing

GTAW: Gas-tungsten-arc-welding

HIP: Hot isostatic pressing

High Pressure Torsion

LAM: Laser additive manufacturing

MA: Mechanical Alloying

PBF: Powder bed fusion

SPS: Spark plasma sintering

SLM: Selective Laser Melting

**Table 1s. Comparison of Experimental and CALPHAD predicted Vickers hardness (HV) for some compositions taken from literature.**

| **Alloys** | **Experimental 1** | **Experimental 2** | **Experimental 3** | **Experimental 4** | **Experimental 5** | **Experimental 6** | **Experimental 7** | **Experimental 8** | **CALPHAD predicted HV@ 300K** |
| --- | --- | --- | --- | --- | --- | --- | --- | --- | --- |
| CoFeNi | Method: Arc melting  C.S: single FCC  Hardness: 125.749 HV  Grain size: --  [25] | Method: Arc melting + homogenization (1473K) + subsequent rolling (92% thickness reduction) + annealing (T=1173K, 1hr)  C.S: single FCC  Hardness: --  Grain size: 28µm  [20] | Method: LENS 750 -AM and CRSA (Cast +homogenized at 1200°C for 1hr + cold rolled (CR) to 90% + solutionized at 850°C for 1 min)  C.S: --  Hardness: --  Grain size: 99µm (LENS); 16µm (CRSA)  Strength: YS=325MPa (LENS); YS=275MPa (CRSA)  [26] | Method: LENS 750 -AM  C.S: single FCC  Hardness: 156 HV  Grain size: --  [27] | -----  -- | -- | -- | -- | 247.48 |
| CoCrNi | Method: Arc melting + homogenization (1000ᐤC, 24h) + subsequent rolling (90% thickness reduction) + annealing (T=1100ᐤC, 5min)  C.S: single FCC  Hardness: --  Strength: YS=250.3Mpa, UTS=759.5Mpa  Elongation: 73.2%  Grain size: --  [28] | Method: Arc melting + homogenization (1473K) + subsequent rolling (92% thickness reduction) + annealing (T=1273K, 1hr)  C.S: single FCC  Hardness: --  Grain size: 41µm  [20] | Method: Arc melting + homogenization (1373K, 48h) + subsequent rolling (88% thickness reduction) + annealing (1073K, 1h; 1123K, 2h; 1173K, 2h)  C.S: single FCC  Hardness: --  Strength:   \|  \| YS  [MPa] \| UTS  MPa] \| \| --- \| --- \| --- \| \| 1073K, 1h \| 523 \| 965 \| \| 1123K, 2h \| 390 \| 851 \| \| 1173K, 2h \| 320 \| 770 \|   Grain size:   \|  \| GS \| \| --- \| --- \| \| 1073K, 1h \| 3.8µm \| \| 1123K, 2h \| 10.9µm \| \| 1173K, 2h \| 21.8µm \|   [29] | Method: Arc melting + homogenization (1200ᐤC, 2h) + subsequent rolling (66% thickness reduction) + annealing (T=1160ᐤC, 3min)  C.S: single FCC  Hardness: --  Strength: YS=430MPa, UTS=900MPa  Grain size: 6.5 µm  [30] | Method: Arc melting + homogenization (at different temperatures, 2h)  C.S: single FCC  Hardness:   \|  \| HV \| \| --- \| --- \| \| 0ᐤC \| 542.5 \| \| 200ᐤC \| 528 \| \| 400ᐤC \| 527 \| \| 500ᐤC \| 531 \| \| 600ᐤC \| 400 \| \| 700ᐤC \| 340 \| \| 800ᐤC \| 245 \| \| 900ᐤC \| 195 \| \| 1000ᐤC \| 173 \| \| 1100ᐤC \| 168 \|   Strength: --  Grain size:   \|  \| GS \| \| --- \| --- \| \| 800ᐤC \| 4.3µm \| \| 900ᐤC \| 15µm \| \| 1000ᐤC \| 42.5µm \| \| 1100ᐤC \| 71µm \|   [31] | Method: Arc melting + homogenization (1473K, 4h) + ECAP (3passes) + annealing (T=300-1000ᐤC, 1hr): PDA (post deformation annealing)  C.S: single FCC  Hardness:   \| No. of passes \| HV \| \| --- \| --- \| \| 0 \| 160±4 \| \| 1 \| 349±5 \| \| 2 \| 446±8 \| \| 3 \| 466±6 \| \| PDA-500ᐤC \| 533 ± 10 \| \| PDA-600ᐤC \| 396 ± 15 \| \| PDA-700ᐤC \| 303 ± 13 \| \| PDA-800ᐤC \| 262 ± 4 \|   Strength:   \|  \| YS  [MPa] \| UTS  MPa] \| \| --- \| --- \| --- \| \| 0 \| 200 \| 486 \| \| 1 \| 829 \| 841 \| \| 2 \| 1068 \| 1140 \| \| 3 \| 1191 \| 1200 \| \| PDA-500ᐤC \| 1298 \| 1501 \| \| PDA-600ᐤC \| 1149 \| 1243 \| \| PDA-700ᐤC \| 607 \| 994 \| \| PDA-800ᐤC \| 528 \| 929 \|   Elongation:   \|  \| % \| \| --- \| --- \| \| 0 \| 78% \| \| 1 \| 19% \| \| 2 \| 9% \| \| 3 \| 9% \| \| PDA-500ᐤC \| 4% \| \| PDA-600ᐤC \| 12% \| \| PDA-700ᐤC \| 45% \| \| PDA-800ᐤC \| 55% \|   Grain size:   \|  \| GS \| \| --- \| --- \| \| 0 \| 120µm \| \| 1 \| 0.290µm \| \| 2 \| 0.162µm \| \| 3 \| 0.126µm \|   [32] | Method: Arc melting + drop casting+ homogenization (1200 °C, 24–48h treatment) +cold forged and cross rolled (~60% thickness reduction) + annealing (800 °C for 1h) +quasi-constrained HPT (7.8GPa pressure)  C.S: single FCC  Hardness: --  Strength:   \|  \| YS  [MPa] \| UTS  MPa] \| \| --- \| --- \| --- \| \| CG \| 402 ±21 \| 784 ±42 \| \| HPT \| 1901 ± 114 \| 2067 ± 153 \| \| 550°C, 10min \| - \| 2170 ± 98 \| \| 500°C, 15h \| 1530 ± 83 \| 1616 ± 38 \| \| 500°C, 100h \| 1452 ± 35 \| 1520 ± 15 \|   Elongation:   \|  \| Elongation (%) \| \| --- \| --- \| \| CG \| 30 ± 2 \| \| HPT \| 3.9 ± 1.5 \| \| 550°C, 10min \| 1.4 ± 0.3 \| \| 500°C, 15h \| 5.2 ± 0.2 \| \| 500°C, 100h \| 10 ± 0.2 \|   Grain size:   \|  \| GS \| \| --- \| --- \| \| 500°C, 10min \| 1.2±0.4µm \| \| 500°C, 15h \| 1.8±0.6µm \| \| 500°C, 100h \| 2.0 ± 0.5 µm \|   [33] | Method: Vacuum induction melting + casting + homogenization (at 1100 °C for 6h, followed by water quenching) + cold rolling (thickness reduction of ~ 80%) + recrystallization (at 900°C for 60 min, followed by water quenching): (initial); + HPT (at 6GPa pressure and 5 turns) + annealing (range of 500–600 °C for 2–60 min)  C.S: single FCC  Hardness:   \|  \| HV \| \| --- \| --- \| \| initial \| 233 \| \| HPT \| 609 \| \| HPT-500ᐤC,15m \| 744 \| \| HPT-500ᐤC,30m \| 750 \| \| HPT-500ᐤC,60m \| 770 \| \| HPT-600ᐤC,2m \| 705 \| \| HPT-600ᐤC,15m \| 587 \|   Strength: --  Grain size: ~ 40–70 nm (in all condition)  [34] | 302.73 |
| FeMnNi [22] | Method: Arc melting + Suction casting + homogenization (1273K, 24h) + subsequent rolling (90% thickness reduction) + annealing (at different temperature and time)  C.S: single FCC   \|  \| HV \| \| --- \| --- \| \| 773K, 1h \| 349±17 \| \| 773K, 2h \| 360±20 \| \| 773K, 4h \| 426±19 \| \| 773K, 8h \| 411±8 \| \| 773K, 16h \| 440±10 \| \| 773K, 24h \| 454±10 \| \| 873K, 1h \| 271±3 \| \| 973K, 1h \| 210±5 \| \| 1073K, 1h \| 215±13 \|   Hardness**:**  **Strength:**   \|  \| YS  [MPa] \| UTS  MPa] \| \| --- \| --- \| --- \| \| 773K, 4h \| 998±11 \| 1144±5 \| \| 773K, 1h \| 994± 24 \| 1073± 43 \| \| 873K, 1h \| 749±5 \| 841± 17 \| \| 1073K, 1h \| 235±3 \| 539±4 \|     Grain size:   \|  \| GS \| \| --- \| --- \| \| 773K,4h \| 0.48 (fine) + 7.15 (coarse) µm \| \| 773K,1h \| 0.44 (fine) + 5.43 (coarse) \| \| 873K,1h \| 0.37(fine)+4.57(coarse) \| \| 1073K, 1h \| 1.91(fine)+10.07(coarse) \|   [35] | Method: High-energy ball-milling+ consolidation by SPS (at 1000ᐤC, 40MPa for 5min in vacuum environment of ~ 6Pa)  C.S: single FCC  Hardness: 342± 8 HV  Strength: YS= 912± 7.5MPa  Elongation: 19± 0.9%  Grain size: 410 nm  [36] | Method: vacuum arc melting of Si-doped (0, 0.5, 1, 2 at% doped) ternary FeMnNi alloys + Suction casting + homogenization (1373K, 24h) + subsequent rolling (90% thickness reduction) + annealed (1273K, 2h)  C.S: single FCC  Hardness: 153 HV (0%Si-FeMnNi)  Strength: YS=178MPa, UTS=428 MPa  Elongation: 45.65%  Grain size: 170 ±10 μm  [37] | Method: Arc melting + Suction casting + homogenization (1000ᐤC, 24h) + subsequent rolling (90% thickness reduction) + Thermal cycling treatment, by cycles of intermittent heat treatment and normalizing at different temperatures and time (500◦C – 15 min – 4 cycles (500◦C TC), 500◦C – 60 min – 1 cycle(500◦C ISO), 600◦C – 15 min – 4 cycles(600◦C TC), 600◦C – 60 min – 1 cycle (600◦C ISO), 700◦C – 1 min – 5 cycles (700◦C TC), 700◦C – 5 min – 1 cycle (700◦C ISO), 800◦C – 1 min – 4 cycles(800◦C TC), and 800◦C – 4 min – 1 cycle (800◦C ISO))  ISO: Isothermal single-step  TC: Thermally cycled  C.S: single phase FCC  Hardness:   \|  \| HV \| \| --- \| --- \| \| 500◦C ISO \| 386 ± 13 \| \| 500◦C TC \| 423 ± 12 \| \| 600◦C ISO \| 297 ± 13 \| \| 600◦C TC \| 340 ± 11 \| \| 700◦C ISO \| 253 ± 9 \| \| 700◦C TC \| 287 ± 16 \| \| 800◦C ISO \| 177 ± 10 \| \| 800◦C TC \| 213 ± 11 \|   Strength:   \|  \| YS[MPa] \| \| --- \| --- \| \| 500◦C ISO \| 998 ± 16 \| \| 500◦C TC \| 1084 ± 13 \| \| 600◦C ISO \| 771 ± 10 \| \| 600◦C TC \| 887 ± 13 \| \| 700◦C ISO \| 610 ± 21 \| \| 700◦C TC \| 798 ± 10 \| \| 800◦C ISO \| 406 ± 11 \| \| 800◦C TC \| 562 ± 10 \|   Grain size:   \|  \| G.S [µm] \| \| --- \| --- \| \| 500◦C ISO \| 0.54μm \| \| 500◦C TC \| 0.43μm \| \| 600◦C ISO \| 2.22μm \| \| 600◦C TC \| 0.65μm \| \| 700◦C ISO \| 3.54μm \| \| 700◦C TC \| 1.07μm \| \| 800◦C ISO \| 10.07μm \| \| 800◦C TC \| 3.51μm \|   [38] | -- | -- | -- | -- | 222.2 |
| CoCrFeNi [22] | Method: Arc melting of CoCrFeNiTa_x_ (x = 0, 0.1, 0.2, 0.3, 0.4, 0.5, and 0.75) + casting  C.S: Single FCC  Hardness: 141 HV  Strength: YS=145 MPa  Elongation: >50%  Grain size: --  [9] | Method: Arc melting (produced ingot): As-solidified sample + homogenization at 1000ᐤC for 24h: Annealed sample  C.S: Single FCC  Hardness: 160±4 (As-solidified); 134±4 (Annealed)  Strength: YS=140 MPa, UTS=488 MPa (As-solidified); YS=130 MPa, UTS=458 MPa (Annealed)  Elongation: 83% (As-solidified); 87% (Annealed)  Grain size: ~100-150 µm [average width] 200-300 µm [average length] (As-solidified); 200 µm (Annealed)  [39] | Method: Mechanical alloying (MA) + spark plasma sintering (SPS) at 900°C+annealing (at 700°C, 800°C, 900°C for 600h)  C.S: FCC+Cr_7_C_3_+Cr_2_O_3_  Hardness:   \|  \| HV \| \| --- \| --- \| \| SPS \| 580±2 \| \| SPS+A-700°C \| 565±2 \| \| SPS+A-800°C \| 520±2 \| \| SPS+A-900°C \| 430±20 \|   Strength: --  Grain size:   \|  \| GS [nm] \| \| --- \| --- \| \| SPS \| 120±28 \| \| SPS+A-700°C \| 128±40 \| \| SPS+A-800°C \| 150±35 \| \| SPS+A-900°C \| 260±75 \|   [40] | Method: Spark plasma sintering of atomized-powder (1050◦C sintering temperature, 100 K/min heating rate, 50 MPa uniaxial sintering pressure, 13min holding time): as-sintered sample + a single ECAP pass (90◦ channel angle, 5mm/min pressing speed): ECAP-processed sample  C.S: Single FCC  Hardness: 192 (as-sintered); 420 (ECAP-processed)  Strength: YS=355 MPa, UTS=654MPa (as-sintered); YS=1032 MPa, UTS=1136 MPa (ECAP-processed)  Grain size: --  [41] | Method: Spark plasma sintering [mixture of atomized CoCrFeNi HEA powder, nickel-coated graphite powder, and nickel-coated MoS2 powder], at 1150 °C temperature, 150 °C/min heating rate and 3 min holding time.  C.S: single FCC (SPSed-CoCrFeNi); FCC+graphite +MoS2 + Ni phase (HEA matrix composite).  Hardness: 238±11 (SPSed-CoCrFeNi HEA); 271±14 (HEA matrix composite).  Microhardness: 395±21 HV (CoCrFeNi HEA powder); 231±23HV (SPSed-CoCrFeNi HEA); 377±17 HV (HEA matrix of the composite)  Strength: YS=387±16 MPa (SPSed-CoCrFeNi HEA); YS= 610±13 (HEA matrix composite).  Grain size: --  [42] | Method: Spark plasma sintering [ mixture of argon-atomized CoCrFeNi HEA powder (80 wt%), Ag powder (10 wt%) and BaF2/CaF2 eutectic powder (10 wt%).] abbreviated as CoCrFeNi-Ag-BaF2/CaF2  C.S: FCC + Ag + BaF2 + CaF2  Hardness: 151 ± 7  Strength: YS=468±8 MPa  Grain size: --  [43] | Method: Arc melting + drop casting CoCrFeNiNb_x_ (x = 0, 0.103, 0.155, 0.206, 0.309 and 0.412)  C.S: FCC CoCrFeNi(0%Nb)  Hardness: --  Strength: YS=147MPa, UTS=413MPa.  Elongation 48%  Grain size: Elongated grains of 150-200µm (average width)  [44] | Method: Powder Bed Fusion (PBF) technology (150 W laser power, 600 mm/s laser speed, 55 μm laser spot diameter, 80μm hatch spacing, and 20μm layer thickness) additively manufactured (AM); Subsequent HPT on AM samples (6.0 GPa compressive pressure, 1 rpm rotational speed for total numbers of revolution, N, of 1/ 2, 1, 2, 4 and 8 turns.)  Vacuum induction melting + drop casting + hot-rolling (1050 ◦C temperature, 64% thickness reduction) + annealing (1100 ◦C, 1 h): conventionally-manufactured (CM)  C.S: Single FCC  Hardness: ~260 (AM); ~160 (CM)  Grain size:   \|  \| GS \| \| --- \| --- \| \| CM \| ~57 ± 20μm \| \| AM+HPT,1turn \| ~105nm \| \| AM+HPT,8turn \| ~90nm \|   [45] | 297 |
| CoCrFeMnNi [23] | Method: Arc melting (produced ingot): As-solidified sample + homogenized at 1000ᐤC for 24h: Annealed sample  C.S: Single FCC  Hardness: 170±4 HV (As-solidified); 135±2 HV (Annealed)  Strength: YS=215MPa, UTS=491MPa (As-solidified); YS=162MPa, UTS=443 MPa (Annealed)  Elongation: 71% (As-solidified); 68% (Annealed)  Grain size: 300-400 µm (As-solidified); ~130 µm (Annealed)  [39] | Method: Arc melting + drop-casting + homogenization at 1273 K for 16 h + HPT under quasi-constrained condition (6.0GPa pressure, 1rpm rotation speed, through total 1, 5 and 10 revolutions). The disks subjected to HPT through 5 rotations were used for PDA at 473–1173 K for total annealing times from 10 to 60 min.  C.S: Single FCC  Hardness:   \|  \| HV \| \| --- \| --- \| \| HPT \| 450 \| \| HPT+PDA-473K, 60m \| 490 \| \| HPT+PDA-673K, 60m \| 515 \| \| HPT+PDA-773K, 60m \| 520 \| \| HPT+PDA-873K, 60m \| 455 \| \| HPT+PDA-973K, 60m \| 355 \| \| HPT+PDA-1073K, 10m \| 290 \| \| HPT+PDA-1073K, 30m \| 245 \| \| HPT+PDA-1073K, 60m \| 220 \| \| HPT+PDA-1173K, 10m \| 170 \| \| HPT+PDA-1173K, 30m \| 155 \| \| HPT+PDA-1173K, 60m \| 140 \|   Strength:   \|  \| YS [MPa] \| UTS[MPa] \| \| --- \| --- \| --- \| \| HPT \| 1400 \| 1740 \| \| HPT+PDA-773K, 60m \| 930 \| 1250 \| \| HPT+PDA-873K, 60m \| 1010 \| 1060 \| \| HPT+PDA-973K, 60m \| 950 \| 1030 \| \| HPT+PDA-1073K, 10m \| 680 \| 830 \| \| HPT+PDA-1073K, 30m \| 570 \| 725 \| \| HPT+PDA-1073K, 60m \| 530 \| 680 \| \| HPT+PDA-1173K, 10m \| 410 \| 630 \| \| HPT+PDA-1173K, 30m \| 390 \| 610 \| \| HPT+PDA-1173K, 60m \| 370 \| 600 \|   Grain size:   \|  \| GS \| \| --- \| --- \| \| HPT \| ~10 nm \| \| HPT+PDA-473K, 60m \| ~15nm \| \| HPT+PDA-673K, 60m \| ~30nm \| \| HPT+PDA-773K, 60m \| ~60 nm \| \| HPT+PDA-873K, 60m \| ~90nm \| \| HPT+PDA-973K, 60m \| ~400nm \| \| HPT+PDA-1073K, 60m \| ~4 µm \| \| HPT+PDA-1173K, 60m \| ~17 µm \|  \|  \| % \| \| --- \| --- \| \| HPT \| 4% \| \| HPT+PDA-473K, 60m \| - \| \| HPT+PDA-673K, 60m \| - \| \| HPT+PDA-773K, 60m \| 5% \| \| HPT+PDA-873K, 60m \| 2% \| \| HPT+PDA-973K, 60m \| 21% \| \| HPT+PDA-1073K, 60m \| 80% \| \| HPT+PDA-1173K, 60m \| 90% \|   [46] | Method: Arc melting + drop-casting + homogenization at 1200K for 48 h + HPT (7.8 GPa pressure, 0.2 rotations/min rotational speed); Specimens subjected to 5 rotations were used for isochronal heat treatments for 1h and isothermal heat treatments at 450ᐤC.  C.S: Single FCC  Hardness:   \|  \| HV \| \| --- \| --- \| \| SPD \| ~520 \| \| Isothermal heat treatment \| 630 \| \| After an annealing time of 100h \| ~910 \|   Strength: --  Grain size: 50 nm (SPD)  [47] | Method: laser-based directed energy deposition (DED) Additive Manufacturing (AM): [as-built]; + annealing (1100°C for 1 h, and furnace cooled at ˜2 × 10−2 K/sec): [annealed].  C.S: Single FCC (both as-built and annealed)  Hardness: --  Strength: YS=424MPa, UTS=651MPa (as-built); YS=232MPa, UTS=647MPa (annealed)  Elongation: 48%(as-built); 58%(annealed)  Grain size: 42μm (as-built); 99μm (annealed)  [48] | Method: Arc melting + drop-casting  C.S: Single FCC  Hardness:176 HV (5N load)  Strength: YS=209MPa, UTS=496MPa  Elongation: 61.7%  Grain size: --  [18] | Method: Electron beam melting  C.S: Single FCC  Hardness: 157.1 HV (10N load)  Strength: YS=205±3, UTS=497±2  Elongation: 63±1  Grain size: ~65µm (average width of the columnar grains)  [49] | Method: laser additive manufacturing (LAM): As-built (AB); + Heat-treatment (laser power of 600 W, at 700 and 1100ᐤC for 4 h) and then furnace cooling to room temperature: Heat-treated (HT)  C.S: Single FCC  Hardness: 200 HV (AB);  Strength:   \|  \| YS  [MPa] \| UTS  MPa] \| \| --- \| --- \| --- \| \| AB \| 346 \| 566 \| \| HT-700ᐤC \| 356 \| 538 \| \| HT-1100ᐤC \| 333 \| 572 \|   Elongation:   \|  \| % \| \| --- \| --- \| \| AB \| ~26% \| \| HT-700ᐤC \| ~42% \| \| HT-1100ᐤC \| ~59% \|   Grain size: AB has relatively smaller grains compared to HTed samples (as grain grows in HTed specimens)[50] | Method: Melted and solidified in magnetic levitation melting furnace (110 mm ingot and 90 mm height) + solution treated (1100°C for 2h) + subsequently hot forged (1000 °C) to a rod (30 mm final diameter) + cold rolled (1 mm final thickness) + annealing.  C.S: Single FCC  Hardness: --  Strength: 888 MPa, 984 MPa (for UFG)  Elongation: 21%  Grain size: 503 ± 181 nm  [51] | 261 |
| Al_0.5_CoCrFeNi | Method: Vacuum arc melting + Forged into plates + homogenization (1423K for 24h) + furnace cooling: base metal (BM); + Gas-tungsten-arc-welded (GTAW) [2-mm arc gap, 40-A current, and 12-V voltage at 80-mm/min weld speed]: GTAW Al_0.5_CoCrFeNi–HEA (TW).  C.S: FCC+BCC (both BM, TW).  Hardness: 280±5HV (BM); 232±10HV (TW)  Strength: YS=380±10 MPa, UTS=810±20MPa (BM); YS=257±15MPa, UTS=689±20MPa (TW)  Elongation: 67±2% (BM); 56±2% (TW).  Grain size: 60µm (BM); (Fine equiaxed grains of 8 to 12µm + elongated grains of lengths 80 to 120µm) (TW). [52] | Method: Arc furnace melting (Ar atmosphere): as-cast; + Aging (in Ar atmosphere for 24 h at temperatures of 350ᐤC, 500ᐤC, 650ᐤC, 800ᐤC or 950ᐤC and then quenched in water): aged samples  C.S: FCC (as-cast); FCC+BCC (aged 350-950ᐤC)  Hardness:   \|  \| HV \| \| --- \| --- \| \| as-cast \| 247±8 \| \| aged-350ᐤC \| 285±8 \| \| aged-500ᐤC \| 275±8 \| \| aged-650ᐤC \| 276±6 \| \| aged-800ᐤC \| 285±2 \| \| aged-950ᐤC \| 255±3 \|   Strength: --  Grain size: --  [53] | Method: Vacuum induction-melting furnace: (as cast); cold rolled (thickness reduction of 80%): cold-rolled + annealing (at 700°C, 800°C, 900°C, 1000°C, 1100°C and 1200°C for 1h): annealed samples.  C.S: FCC+BCC  Hardness: 180HV (as-cast); 480HV (cold-rolled)  Strength:   \|  \| YS  [MPa] \| UTS [MPa] \| \| --- \| --- \| --- \| \| as-cast \| 402 \| 568 \| \| cold-rolled \| 1396 \| 1461 \|   Elongation: 33.68% (as-cast); 5.37% (cold-rolled)  G.S: --  [54] | Method: Vacuum arc melting + casting: (As-cast) + homogenization at 1100ᐤC for 24h: (Homogenized) + cold-rolled by 60 % in reduction: (Cold-rolled) + annealing at 1100ᐤC for 1h, followed by air-cooling (Annealed)  C.S: FCC+BCC  Hardness:   \|  \| HV \| \| --- \| --- \| \| As-cast \| 155±20 \| \| Homogenized \| 230±12 \| \| Cold-rolled \| 425±15 \| \| Annealed \| 250±10 \|   Strength:   \|  \| YS  [MPa] \| UTS [MPa] \| \| --- \| --- \| --- \| \| As-cast \| 294 ±9 \| 367 ±12 \| \| Homogenized \| 319 ±10 \| 468 ±11 \| \| Cold-rolled \| 545 ±16 \| 834 ±13 \| \| Annealed \| 432 ±14 \| 569 ±9 \|   Elongation:   \|  \| % \| \| --- \| --- \| \| As-cast \| 23% \| \| Homogenized \| 30% \| \| Cold-rolled \| 26% \| \| Annealed \| 28% \|   Grain size: --  [55] | Method: Selective Laser Melting (SLM) [200 W laser power, 90μm scanning pitch, 40μm layer thickness and 0.8 m/s scanning speed, 67◦ laser rotation between each layer]: SLM-ed; + heat treatment (1073K, 1173K, 1273K, 1373K and 1673K for 4h + subsequent cooling down to the room temperature with the furnace: (heat-treated).  C.S: FCC (SLM-ed); FCC+BCC (heat-treated)  Hardness:   \|  \| HV \| \| --- \| --- \| \| untreated \| 265 \| \| 1073K \| 441 \| \| 1173K \| 338 \| \| 1273K \| 400 \| \| 1373K \| 295 \| \| 1673K \| 287 \|   Strength: YS=339MPa, UTS=563MPa (heat-treated 1073K)  Grain size: --  [56] | Method: Induction-melting + casting: (as-cast) + cold-rolling (thickness reduction of 70%): cold-rolled + annealing (at 1200◦C for 1h followed by water quenching): denoted as CRSA + Aging( CRSA samples were aged at 850 ◦C for 1, 4, 8, and 16 h: abbreviated as CRSA–850◦C/1 h, CRSA–850◦C/4 h, CRSA–850◦C/8 h and CRSA–850◦C/16 h); cold-rolled samples were directly aged at 800 ◦C and 900◦C for 1h, which are named as CR–800◦Cand CR-900◦C.  C.S: FCC(CRSA); FCC+BCC+B2 (CRSA–850◦C/4-16h)  Hardness:   \|  \| HV \| \| --- \| --- \| \| CRSA \| 194 \| \| Aged-850◦C, 1h \| 215 \| \| Aged-850◦C, 4h \| 240 \| \| Aged-850◦C, 8h \| 275 \| \| Aged-850◦C, 16h \| 298 \|   Strength: --   \|  \| YS  [MPa] \| UTS [MPa] \| \| --- \| --- \| --- \| \| CRSA \| 321 \| 762 \| \| CRSA-850◦C/16 h \| 514 \| 975 \| \| CR–900 ◦C \| 891 \| - \| \| CR–800◦C \| 1114 \| - \|   Elongation:   \|  \| (%) \| \| --- \| --- \| \| CRSA \| 49.6% \| \| CRSA–850◦C/16 h \| 26.2% \| \| CR–900◦C \| 12.4% \| \| CR–800◦C \| 7.2% \|   Grain size: --  [57] | Method: Arc melting + casting + heat-treatment (600ᐤC/51h, 600ᐤC/17d, 600ᐤC/22d, 600ᐤC/30d, 600ᐤC/43d).  C.S: FCC [DC-matrix] (as-cast); FCC [DC-matrix] + L12[nano-particles] (600ᐤC/51h); FCC[DC-matrix] + L12[tweed morphology] (600ᐤC/17d); FCC[DC-matrix] + L12[tweed morphology] (600ᐤC/22d); FCC[DC-matrix] + B2[cellular morphology] (600ᐤC/30d); FCC[DC-matrix] + B2[partial globular morphology] (600ᐤC/43d);  Hardness:   \|  \| HV \| \| --- \| --- \| \| as-cast \| ~235 \| \| 600ᐤC/51h \| ~234 \| \| 600ᐤC/17d \| ~413 \| \| 600ᐤC/22d \| ~417 \| \| 600ᐤC/30d \| ~415 \| \| 600ᐤC/43d \| ~411 \|   Strength: --  Grain size: --  [58] |  | 349.56 |
| HfNbTaTiZr | Method: Vacuum arc melting + casting (Discs with 20 mm diameter and 1 mm thickness) + HPT (2.5GPa pressure, strained by various numbers of revolutions N=0 (without any torsion straining),1/4, 1/2, 1, 5, 15))  Crystal structure: BCC (as-cast)  Hardness:   \|  \| HV \| \| --- \| --- \| \| As-cast \| ~344 \| \| strain[e]> 2, stage 1 \| ~390 \| \| strain[e]>50, stage 2 \| ~490 \| \| strain[e]> 100, stage 3 \| ~510 \|  \|  \| YS  [MPa] \| UTS [MPa] \| \| --- \| --- \| --- \| \| as-cast \| 1030±50 \| 1070±30 \| \| strain[e]> 50, stage 2 \| 1480±50 \| 1670±40 \|   Strength:    Elongation: 15±3% (as-cast); 10% (strain[e]> 50, stage 2)  Grain size: 200mm(as-cast); ~80nm (strain[e]> 50); Further increase of strain did not lead to an additional reduction of the grain size.  [59] | Method: Vacuum arc melting + homogenization (1200ᐤC, 24h) + annealing (600, 800 and 1000ᐤC for 1– 100h)  Crystal structure: BCC (homogenized); BCC+HCP (annealed)  Hardness:   \|  \| HV \| \| --- \| --- \| \| Homogenized \| 370 \| \| A-600ᐤC,1h \| 470 \| \| A-600ᐤC,10h \| 500 \|   Strength: --  G.S: 185±75 µm (Homogenized); 25nm(A-600,10h)  [60] | Method: Vacuum-arc melting + casting (~10mm thickness and ~50mm diameter button) + hot- isostatic pressing [207MPa for 2h]: (HIP'ed) + annealing (at 1200ᐤC for 24h) + Flat (sheet) rolling (65% and 86.4% thickness reduction): C.R(65% and 86.4%) + annealing (2h at 800ᐤC, 1000ᐤC, and 1200ᐤC): (CR+A)  Crystal structure:   \|  \| CS \| \| --- \| --- \| \| C.R \| BCC \| \| C.R+A(800ᐤC) \| BCC1+BCC2 \| \| C.R+A(1000ᐤC) \| BCC \| \| C.R+A(1200ᐤC) \| BCC \|   Hardness:   \|  \| HV \| \| --- \| --- \| \| before rolling \| 360 ± 10 \| \| C.R(65%) \| 367 ± 8 \| \| C.R(86.4%) \| 366 ± 4 \| \| C.R(65%) + A(800ᐤC) \| 374 ± 8 \| \| C.R(86.4%) + A(800ᐤC) \| 375± 5 \| \| C.R(65%) + A(1000ᐤC) \| 347 ± 6 \| \| C.R(86.4%) + A(1000ᐤC) \| 351 ± 7 \| \| C.R(65%) + A(1200ᐤC) \| 367 ± 5 \| \| C.R(86.4%) + A(1200ᐤC) \| 370 ± 5 \|   Strength:   \|  \| YS  [MPa] \| UTS [MPa] \| \| --- \| --- \| --- \| \| C.R \| 1202 \| 1295 \| \| C.R + A(800ᐤC,2h) \| 1303 \| 1334 \| \| C.R + A(1000ᐤC,2h) \| 1145 \| 1262 \|   Elongation:   \|  \| % \| \| --- \| --- \| \| C.R \| 4.7% \| \| C.R+A (800ᐤC, 2h) \| 1.9% \| \| C.R+A (1000ᐤC, 2h) \| 9.7% \|   Grain size:   \|  \| GS \| \| --- \| --- \| \| Cast + Annealed \| ~140  mm \| \| C.R 65% + A (1000ᐤC/2 h) \| 35±5 µm \| \| C.R 86.4% + A (1000ᐤC/2 h) \| 22±3 µm \| \| C.R 86.4% + A (1200ᐤC/2h \| 76 µm \|   [61] | Method: Mechanical alloying + spark plasma sintering [SPS] (1000ᐤC temperature, 50 MPa pressure) + Electro spark deposition on stainless steel substrate (electrodes cut from bulk material)  Crystal structure: BCC+FCC (small amount)  Hardness:   \|  \| HV \| \| --- \| --- \| \| Substrate (316 SS) \| 250±27.56 \| \| HfNbTaTiZr-HEA sintered \| 840±67.6 \| \| HfNbTaTiZr-HEA coating \| 254±28.2 \|   Strength: --  Grain size: --  [62] | Method: Melting using plasma arc furnace+ casting (ingots were annealed just below the melting point for 14h) + solutionization (1473K for 1h followed by water cooling): ST single crystal;  Master ingots were homogenized at 1773 K for 5h followed by furnace cooling + polycrystal solutionization (at 1473 K for 1h, followed by water quenching): ST-polycrystal;  In addition, some specimens were cold rolled to 70% + annealing (at 673–1173K for 1–24h): C.R + A  Crystal structure: BCC  Micro-Hardness:   \|  \| HV \| \| --- \| --- \| \| ST-polycrystals \| 307 \| \| ST-polycrystals +A-673K \| 466 \| \| ST-polycrystals +A-773K \| 526 \| \| ST-polycrystals +A-873K \| 428 \| \| ST+C.R+A-1073K \| 300 \|   Strength:   \|  \| YS[MPa] \| \| --- \| --- \| \| ST single crystal-273K \| 925 \| \| ST single crystal-673K \| 541 \| \| ST single crystal-873K \| 1105 \| \| ST single crystal-1073K \| 496 \| \| ST-polycrystal-273K \| 1031 \| \| ST-polycrystal-673K \| 658 \| \| ST-polycrystal-873K \| 708 \| \| ST-polycrystal-1073K \| 507 \|   Strain rate= 1.7 × 10^−4^/s  Grain size: --  [63] | Method: Powder metallurgy (consisting of mixing) + cold isostatically pressing (CIP) using a pressure of 400MPa + sintering (at 1400◦C, with 20◦C/min heating rate for 2, 4, 8, 16, 32 and 64h): sintered + annealing (at 1200◦C for 1h, subsequent water quenching): “1200 ◦C/1h/WQ”  Crystal structure: BCC+BCC2+HCP (sintered,64h); BCC+HCP (sintered,64h+1200 ◦C/1h/WQ);  Hardness:   \|  \| HV \| \| --- \| --- \| \| sintered,2h \| 295 \| \| sintered,4h \| 296 \| \| sintered,8h \| 302 \| \| sintered,16h \| 337 \| \| sintered,32h \| 330 \| \| sintered,64h \| 391 \| \| sintered,2h +1200 ◦C/1h/WQ \| 308 \| \| sintered,4h +1200 ◦C/1h/WQ \| 300 \| \| sintered,8h +1200 ◦C/1h/WQ \| 320 \| \| sintered,16h +1200 ◦C/1h/WQ \| 310 \| \| sintered,32h +1200 ◦C/1h/WQ \| 330 \| \| sintered,64h +1200 ◦C/1h/WQ \| 324 \|   Strength: --  Grain size: 25µm (sintered 1400◦C,2h); 60µm (sintered 1400◦C,64h);  [64] | Method: Vacuum arc melting + casting + annealing (1200°C for 24 h): initial state; + Unidirectional multipass rolling (at room temperature using a fixed rolling speed of 30 mm/s to a thickness strain (ε_th_) of 5, 15, 25, 40, 60, and 80%)  Crystal structure: Single BCC  Hardness:   \|  \| HV \| \| --- \| --- \| \| initial state \| 323 \| \| ε_th_ =15% \| 364 \| \| ε_th_ =80% \| 394 \|   Strength: YS=1220MPa, UTS=1320MPa (ε_th_=80%)  Elongation: 3.4% (ε_th_=80%)  Grain size:200±90μm (initial state)  [65] |  | 428.86 |
| Al0.1CoCrFeNi | Method: Arc-melting + Casting + hot-isostatic pressing (at 1473 K, 100 MPa for 4 h): HIPed + High pressure torsion (HPT) (6 GPa pressure, N=1 and 2, r=4mm, ε_N=1_ =3.72, and ε_N=2_ =4.52).  C.S: Single FCC  Hardness: 135HV (HIPed); 482 HV (HPT, N=2)  Strength: --  Grain size: several mm (HIPed); ~80nm (HPT, N=2, near the edge)  [66] | Method: As-cast + dynamic equal channel angular pressing (D-ECAP): D-ECAP-processed + annealing (at 500◦C, 700◦C or 900◦C for 1h in a vacuum furnace): annealed  C.S: Single FCC  Hardness: --  Strength:   \|  \| YS  [MPa] \| UTS [MPa] \| \| --- \| --- \| --- \| \| As-cast \| 249 \| 505 \| \| D-ECAP \| 635 \| 741 \| \| D-ECAP+500◦C annealing \| 559 \| 678 \| \| D-ECAP+700◦C annealing \| 403 \| 602 \| \| D-ECAP+900◦C annealing \| 346 \| 587 \|   Elongation:   \|  \| % \| \| --- \| --- \| \| As-cast \| 54% \| \| D-ECAP \| 16% \| \| D-ECAP+500◦C annealing \| 18% \| \| D-ECAP+700◦C annealing \| 25% \| \| D-ECAP+900◦C annealing \| 42% \|   Grain size: ∼20μm (D-ECAP+900◦C annealing)  [67] | Method: Unidirectional rolling (at room temperature for thickness reductions of 20% and 40%); annealing of 40% cold-worked plates (550 °C/24 h, 620 °C/50 h, 700 °C/5h and 800 °C/1h)  C.S: Single FCC  Hardness: --  Strength:   \|  \| YS  [MPa] \| UTS [MPa] \| \| --- \| --- \| --- \| \| As-cast \| 175 \| 880 \| \| 20% CR \| 464 \| 563 \| \| 40% CR \| 749 \| 824 \| \| 40% CR + 550°C/24h (Recovery) \| 552 \| 802 \| \| 40% CR + 620°C/50h (Recovery) \| 412 \| 892 \| \| 40% CR + 700°C/5h (Partial Recrystallization) \| 520 \| 779 \| \| 40% CR + 800°C/1h (Partial Recrystallization)) \| 360 \| 790 \|   Elongation:   \|  \| % \| \| --- \| --- \| \| As-cast \| 64% \| \| 20% CR \| 23% \| \| 40% CR \| 14% \| \| 40%CR + 550 °C/24h (Recovery) \| 42% \| \| 40%CR + 620 °C/50h (Recovery) \| 40% \| \| 40%CR + 700 °C/5h (Partial Recrystallization) \| 31% \| \| 40%CR + 800 °C/1h (Partial Recrystallization) \| 39% \|   **[68]** | Method: Vacuum induction melting + Casting (10mm in diameter and 1mm in thickness disc-shaped samples) + homogenization (1273K for 1h + quenching in iced water): initial sample; HPT (6 GPa pressure, for N = 1/16, 1/8, 1/4, 1/2, 1, 5, and 10 turns with a rotation speed of 1 rpm at room temperature)  C.S: Single FCC  Hardness: 155 (initial sample); ~520 (HPT, N=1)  Strength:   \|  \| YS [MPa] \| \| --- \| --- \| \| Initial sample \| 250 \| \| HPT, N=1/16 \| 1300 \| \| HPT, N=1/8 \| 1360 \| \| HPT, N=1/4 \| 1520 \| \| HPT, N=1/2 \| 1770 \| \| HPT, N=1 \| 1960 \| \| HPT, N=5 \| 1950 \| \| HPT, N=10 \| 1900 \|   Elongation: 87% (initial sample)  Grain size: ~25 µm (initial sample); 29nm (HPT, N=10)  **[69]** | Method: Arc-melting + drop-casting; Cold-rolling (~3.3 mm/s rolling speed, thickness reduction from ~2 mm to ~0.5mm) + annealing (at 673, 873, 1,073, and 1273 K for 1 h): C.R + A-  C.S: Single FCC  Hardness: --  Strength:   \|  \| YS [MPa] \| \| --- \| --- \| \| C.R \| ~1233 ±35 \| \| C.R + A-673K \| ~1396 ±23 \| \| C.R + A-873K \| ~746 ± 46 \| \| C.R + A-1073K \| ~364±14 \| \| C.R + A-1273K \| ~210±4 \|   Elongation:   \|  \| % \| \| --- \| --- \| \| C.R \| ~1.4 ± 0.2% \| \| C.R + A-673K \| ~1.1 ± 0.1% \| \| C.R + A-873K \| ~28 ± 2.5% \| \| C.R + A-1073K \| ~38.3 ± 1.9% \| \| C.R + A-1273K \| ~40.5 ± 1.7% \|   Grain size: ~8μm (C.R + A-1073K); ~88μm (C.R + A-1273K)  [70] | Method: Vacuum induction melting + casting (referred to as-received) + friction stir processing (FSP)  C.S: Single FCC  Hardness: --  Strength: 160 ± 7MPa, 389 ± 42MPa (as-received); 544 ± 50MPa, 730 ± 19MPa (FSP)   \|  \| YS  [MPa] \| UTS [MPa] \| \| --- \| --- \| --- \| \| as-received \| 160 ± 7 \| 389 ± 42 \| \| FSP \| 544 ± 50 \| 730 ± 19 \|   Elongation: 44 ± 15%(as-received); 27 ± 1%(FSP)  Grain size: ∼1000–2000µm (as-received); ∼3–14µm (FSP)  [71] |  |  | 247.54 |

**2. Description of Tree-based ML regressors**

- 1. **Decision Tree Regressor (DTR)**

Decision tree is one tree structure which is based on if-else loop.


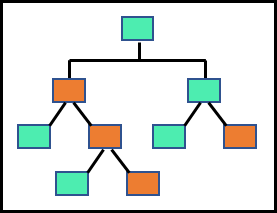


**Figure 2.1s.** Workflow of Decision Tree Regressor

- 1. **Random Forest Regressor (RFR)**

RFR is an ensemble of various decision tree, based on bagging technique, where each tree is trained on a random subset of data and random subset of features. The final prediction is obtained by taking the average of predictions of each individual tree.


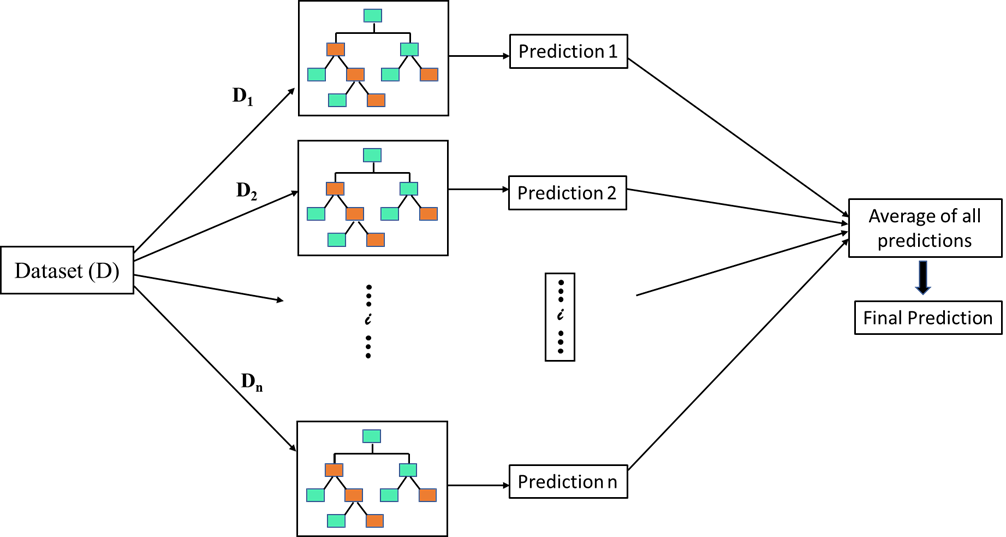


**Figure 2.2s.** Workflow of Random Forest Regressor

- 1. **Gradient Boosting Regressor (GBR)**

GBR is also an ensemble of various decision tree, but it is based on boosting method. It combines multiple weak learners (typically decision trees) and build them sequentially where each subsequent tree tries to rectify the errors made by previous tree, thus create a robust predictive model.


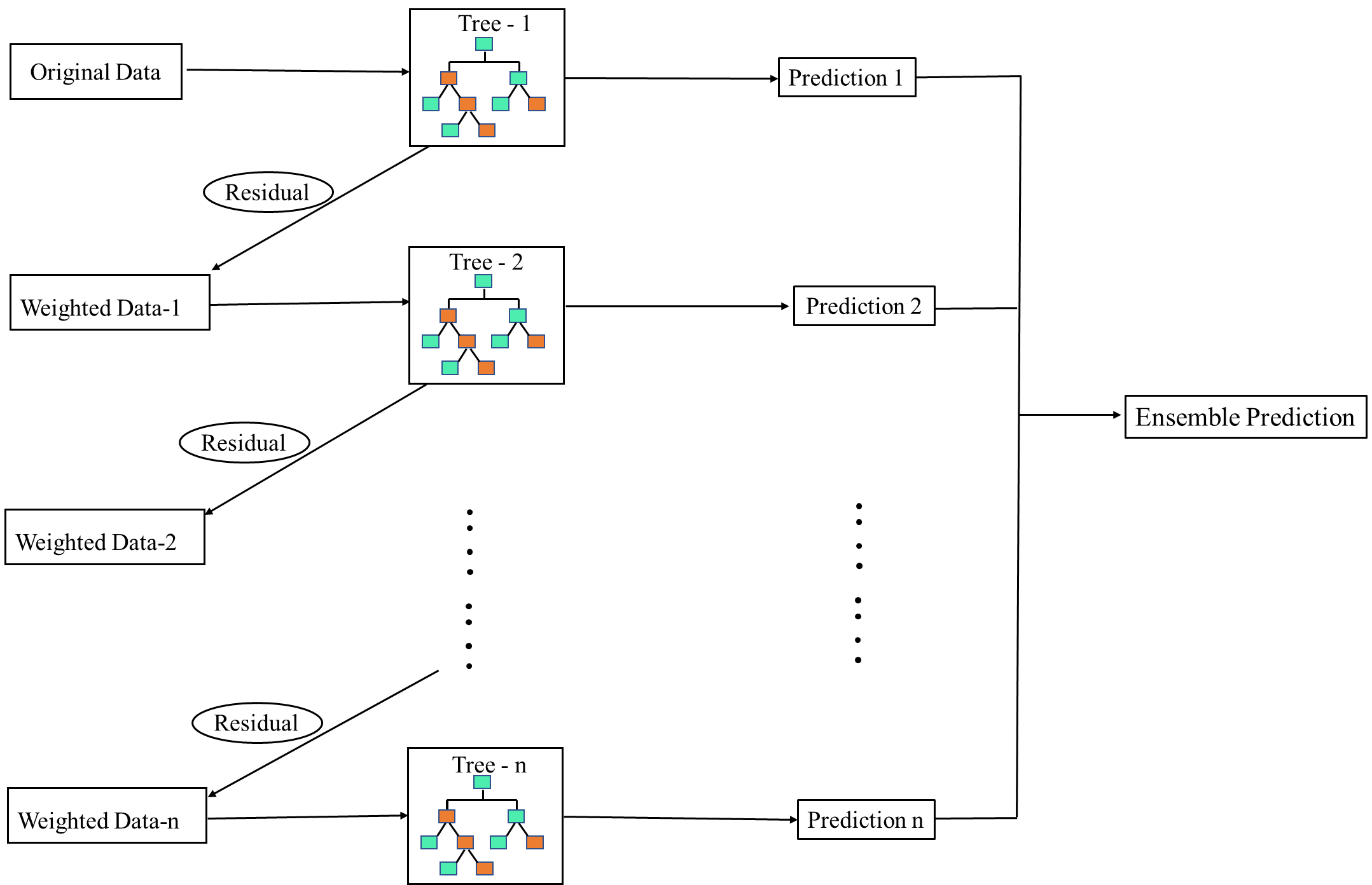


**Figure 2.3s.** Workflow of Gradient Boosting Regressor

- 1. **XGBoost Regressor (XGBR)**

XGBoost (Extreme Gradient Boosting) is an optimized implementation of Gradient Boosting, which incorporates additional regularization, parallel processing and tree pruning. It is highly efficient and perform better compared to traditional Gradient Boosting.


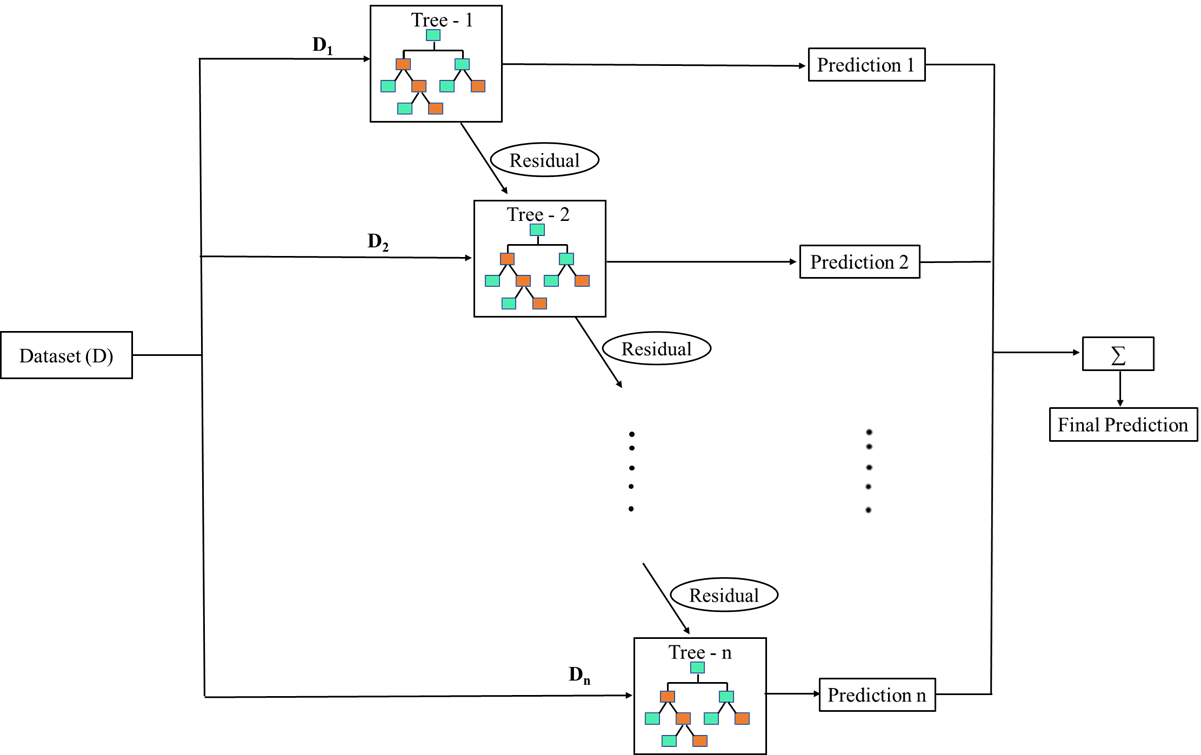


**Figure 2.4s.** Workflow of XGBoost Regressor

- 1. **AdaBoost Regressor (ABR)**

ABR is also an ensemble learning algorithm but it is created based on decision stump. Decision stumps are nothing but decision trees with one node and two leaves. RF is based on multiple decision trees having different depths.


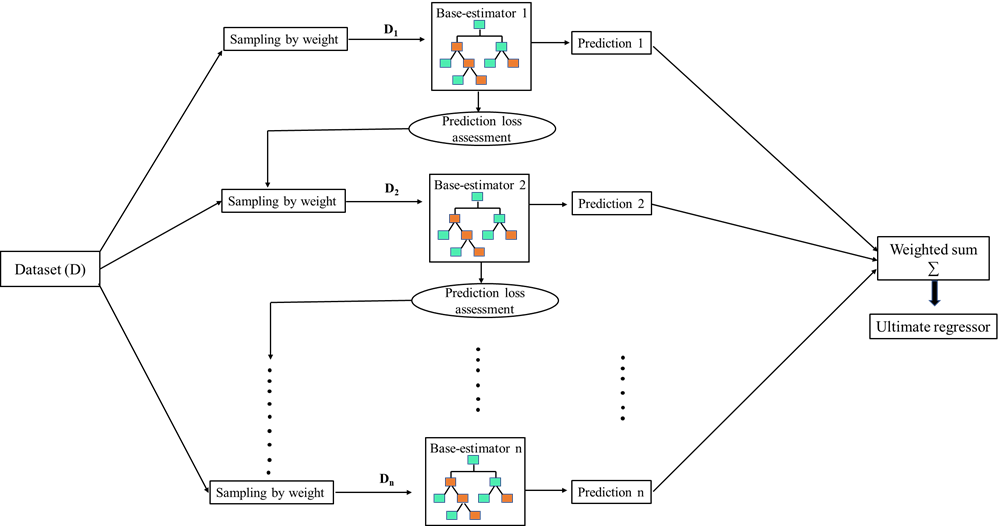


**Figure 2.5s.** Workflow of AdaBoost Regressor

**2.6 Extra Trees Regressor (ETR)**

ETR also known as Extremely Randomized trees is an ensemble of various decision trees. It is quite similar to RFR as based on bagging method except for the random selection of split values.


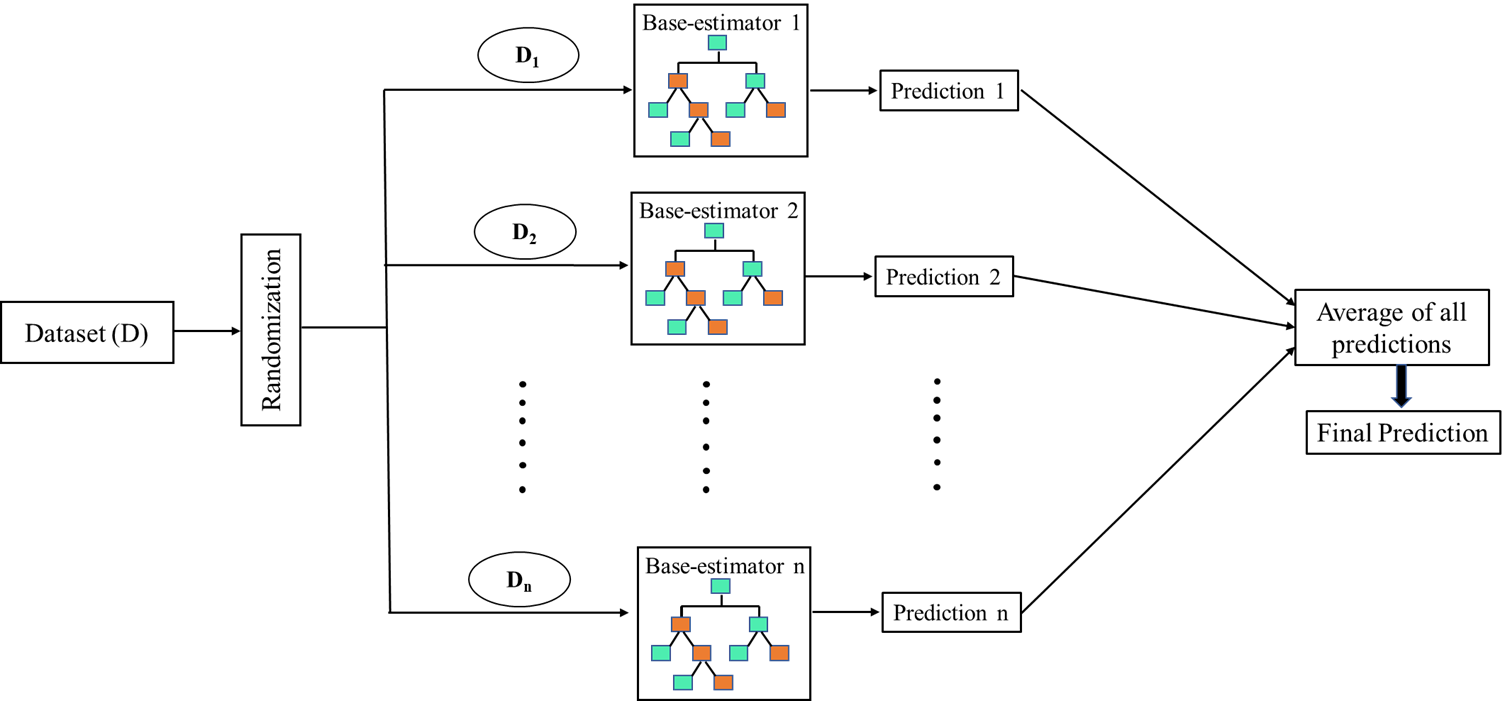


**Figure 2.6s.** Workflow of Extra Trees Regressor

**Table 1:** Summary of the optimized hyperparameters for the chosen ML algorithms.

| **Regression algorithm** | **Hyperparameters** |
| --- | --- |
| Decision Tree Regressor (DTR) | min_samples_split: 4;  min_sample_leaf: 3;  max_features: None |
| Random Forest Regressor (RFR) | n_estimators: 651;  min_samples_split: 2;  min_sample_leaf: 1;  max_depth: 70;  bootstrap: True  max_features: 'sqrt' |
| Gradient Boost Regressor (GBR) | Subsample: 0.97;  n_estimators: 609;  min_samples_split: 9;  min_sample_leaf: 3;  max_depth: 8;  learning_rate: 0.042  max_features: 'log2' |
| AdaBoost Regressor (ABR) | learning_rate: 0.78;  loss: linear;  n_estimators: 280 |
| XGBoost (XGB) | colsample_bytree: 0.87;  gamma: 4.7;  learning_rate: 0.22;  max_depth: 7;  min_child_weight: 5;  n_estimators: 1738;  subsample: 0.94 |
| Extra Trees Regressor (ETR) | n_estimators: 1301;  min_samples_split: 4;  min_sample_leaf: 1;  max_depth: None;  bootstrap: False  max_features: 'log2' |

**3. Optimization techniques**

Description of various optimization techniques used in this paper is elaborated in this section.

We obtained several new compositions by enforcing composition constraint such that the sum of 3, 4, 5, 6,7, 8, 9, or 10 elements become 100.

- 1. **Genetic Algorithm**

Genetic algorithm is one of the most popular evolutionary algorithms based on the principle of Darwin’s theory of survival of the fittest. It includes random initialization of population, selection, crossover, mutation to generate new population with improved fitness. In this method, the variables (or elements in particular for our objective) corresponds to the chromosomes of population, and based on the best fitness value (i.e., the best hardness value), they get selected for recombination and mutation to generate new population with better fitness for the next generation (i.e, compositions corresponding to higher hardness than the previous generation), and the optimal or near-optimal solution obtained after several number of generations represents the best solution of the problem. Complete description of working principle of genetic algorithm is shown in Figure 3.1s.


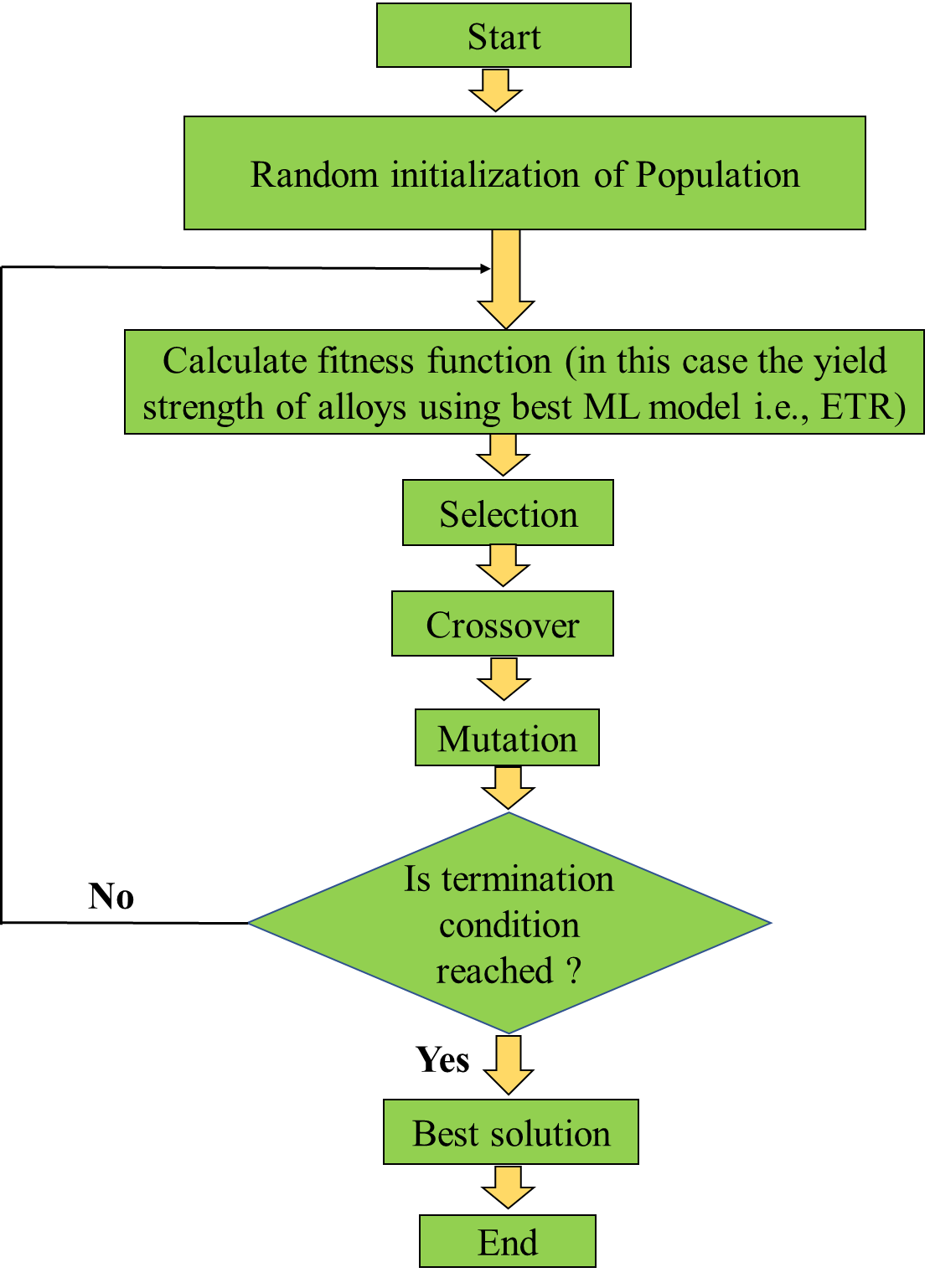


**Figure 3.1s.** Flowchart of working principle of Genetic Algorithm.

| **GA Parameters** | **Values** |
| --- | --- |
| Population size | Range of 50-1000. |
| Crossover rate | Range of 0.6 - 0.9. |
| Mutation rate | Range of 0.01 - 0.1. |
| Selection method | Tournament wheel selection. |
| Number of generations | Range of 1000-5000. |

- 1. **Particle Swarm Optimization**

Particle swarm optimization (PSO) technique is a nature-inspired technique based on social behavior of bird flocking and fish schooling. It is a robust stochastic optimization technique based on the movement and intelligence of swarms, useful to solve difficult optimization problems. Here, each particle represents a potential solution, which move in search space based on individual and group experience. Each particle adjusts its position by considering its personnel best-known position and global best position. One notable drawback of swarm algorithms is the need for subjective parameter tuning. Incorrect parameter setting can cause the algorithm to converge prematurely [93], resulting in suboptimal solutions. An effective approach to address this issue is to find methods that reduce or, if possible, eliminate the need for parameter tuning. Complete description of working principle of particle swarm optimization algorithm is shown in Figure 3.2s.


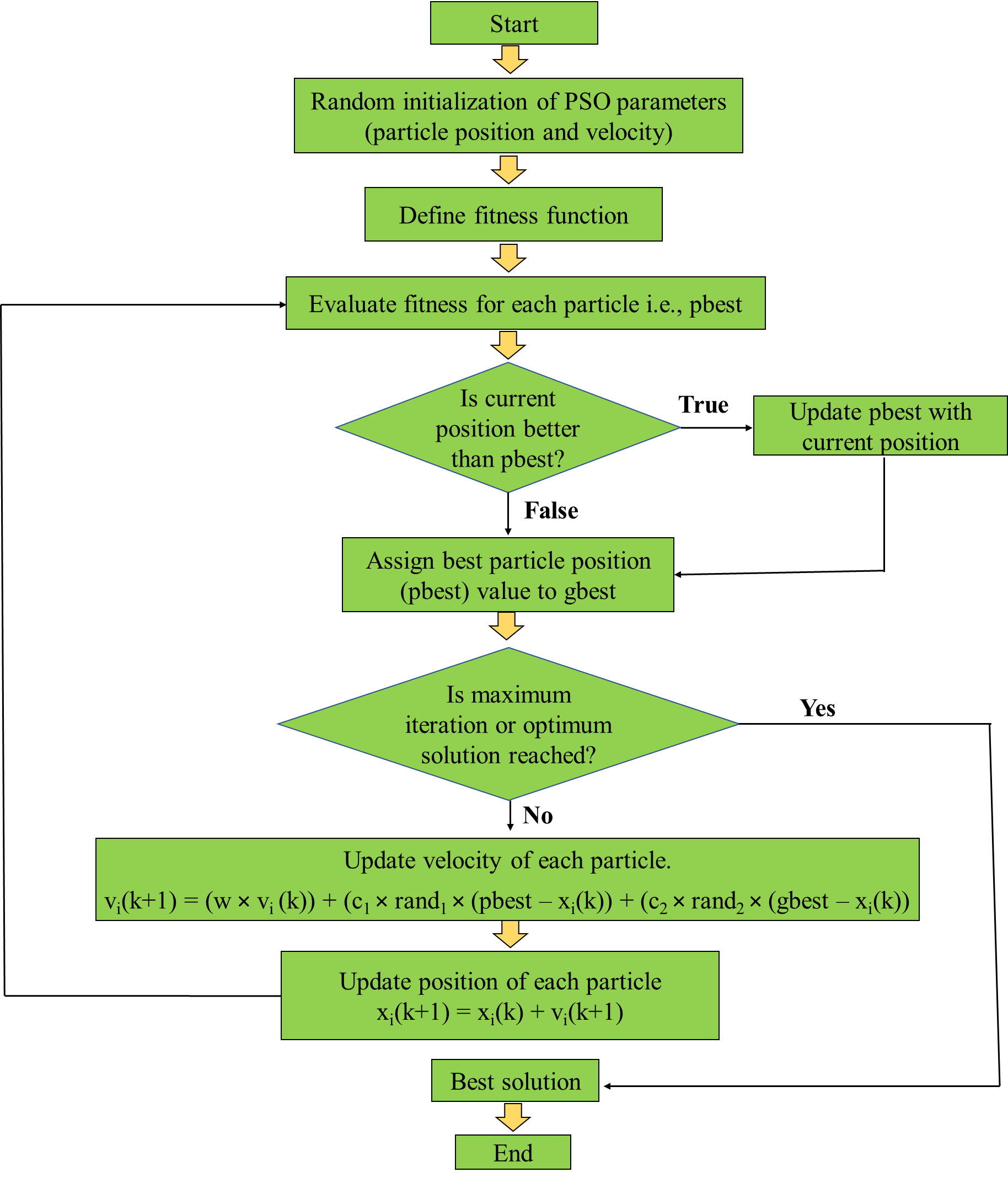


**Figure 3.2s.** Flowchart of working principle of Particle swarm optimization.

| **PSO Parameters** | **Values** |
| --- | --- |
| Swarm size (population size) | Range of 50-100. |
| Cognitive coefficient (c1) | Range of 1.5 - 2.0. |
| Social coefficient (c2) | Range of 1.5 - 2.0. |
| Inertia weight (w) | Range of 0.7 - 1.5. |
| Maximum number of iterations | Range of 100-5000. |

- 1. **Whale optimization Algorithm**

WOA is inspired by the foraging behaviour of Humpback whales. Humpback whales hunt schools of krills or small fishes close to the surface by creating distinctive bubbles along a spiral path and this strategy is known as bubble-net hunting strategy of humpback whales. The working principle of WOA is shown in Figure 3.3s.


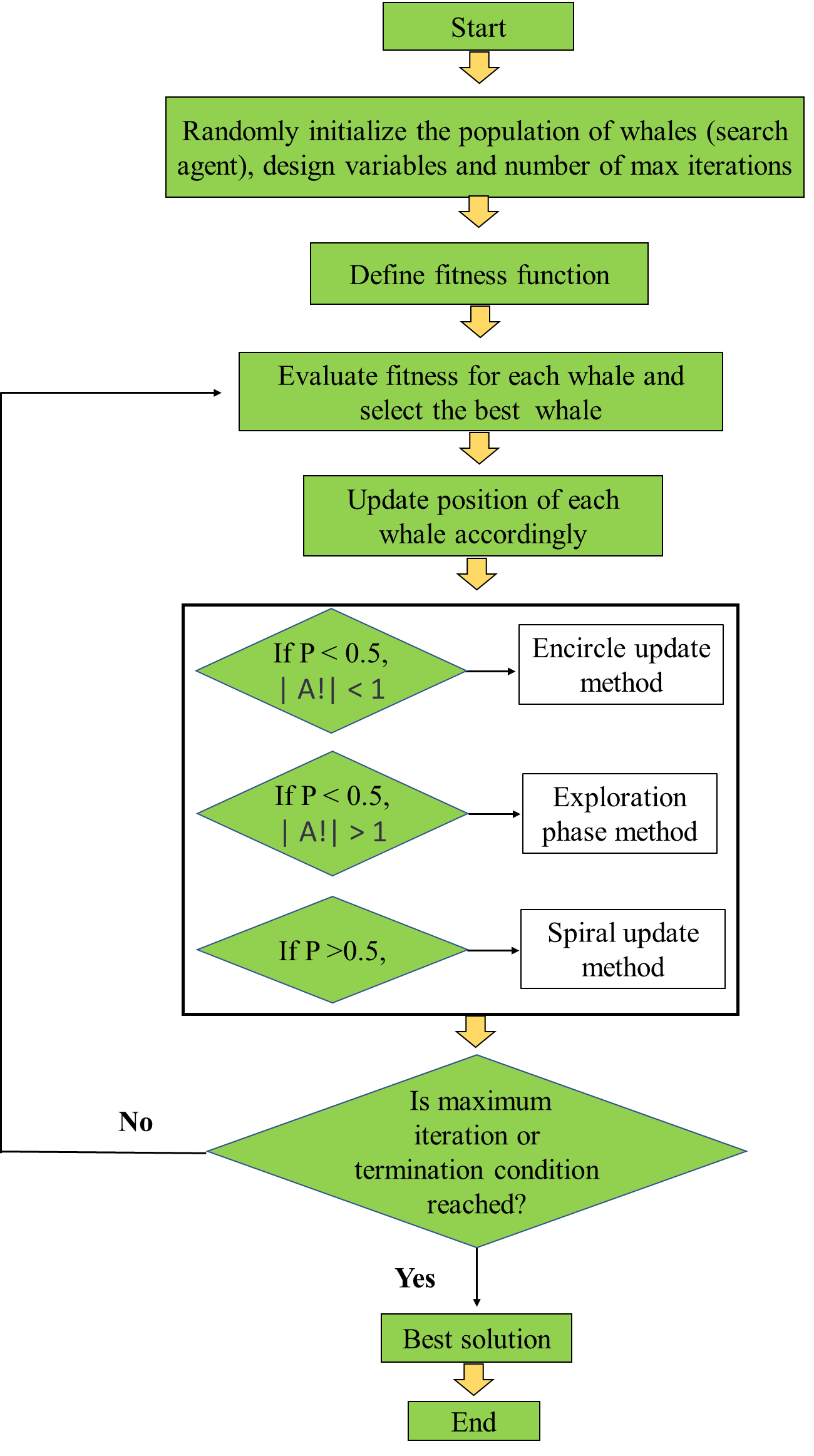


**Figure 3.3s.** Flowchart of working principle of Whale optimization Algorithm.

| **WOA Parameters** | **Values** |
| --- | --- |
| Population size (Number of whales) | Range of 20-300. |
| Exploration and exploitation control parameter (a) | Linearly decreasing from 2 to 0 during iterations. |
| Convergence control parameter (b) | Range of 0.5-1.0. |
| Maximum number of iterations | Range of 20-1000. |

- 1. **Ant Colony optimization**

Ant colony optimization is based on the cooperative behavior of real ant colonies which enables them to find the shortest path from the nest to the food source. Ants are social insects and as many as several millions live together in a colony or nest or anthill. They use pheromone as a means of communication. The ant colony optimization mimics the foraging behavior of ants. Initially, ants wander randomly in different directions. Once any one or more ants find food source, they return to their colony (with food) while leaving pheromone trails. The pheromone is made of certain chemicals produced by a living organism to send messages or signals to other members of the same species. If other ants find such a path, they follow the trail to the food source instead of wandering randomly. When they return to their colony, they too leave pheromone reinforcing the existing pheromone intensity. With time, pheromone evaporates, thus reducing the strength of the pheromone. Eventually, the ants adjust and find the shortest path to the food source. The working principle of ant colony optimization algorithm is shown in Figure 3.4s.

**
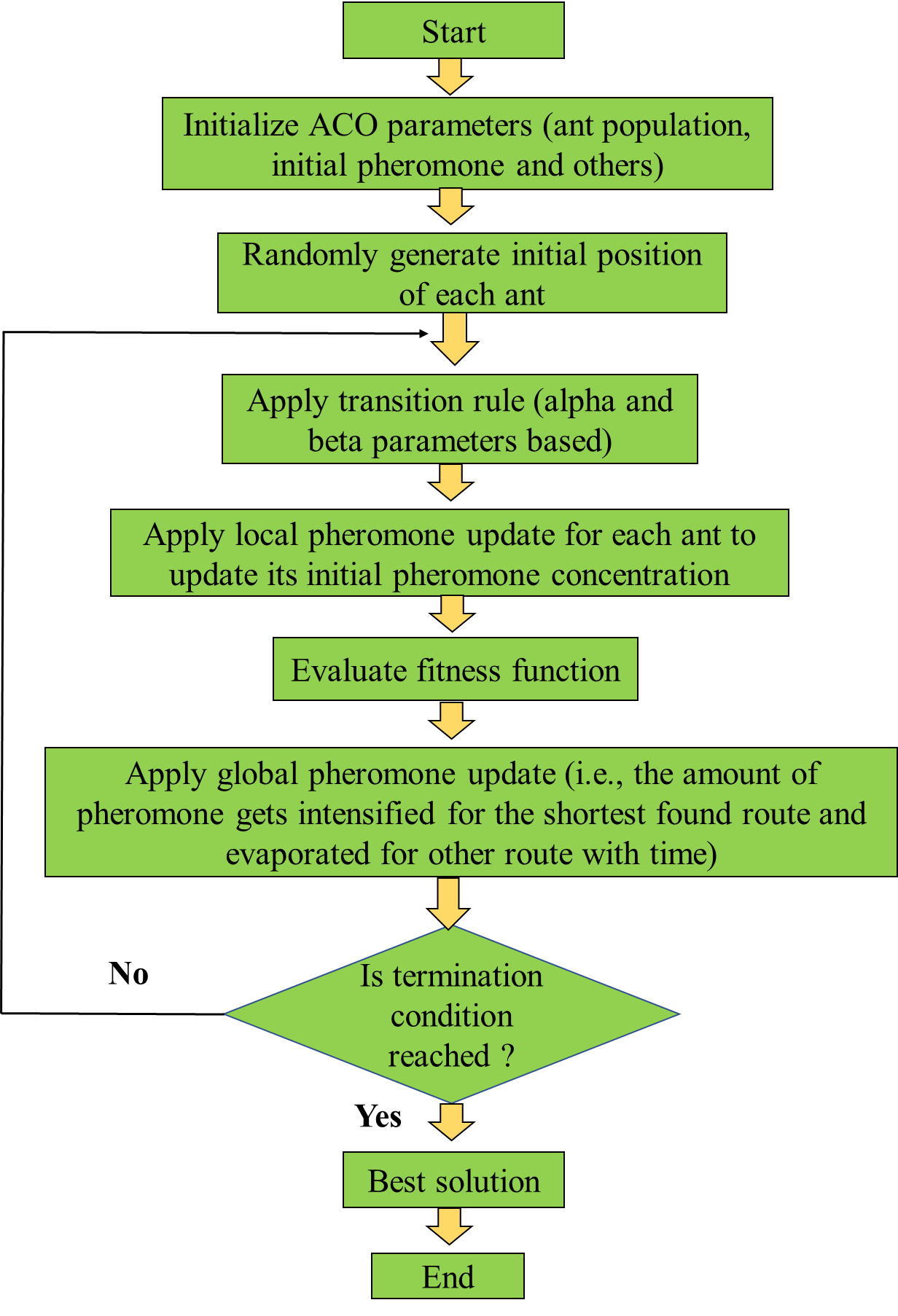
**

**Figure 3.4s.** Flowchart of working principle of Ant Colony optimization.

| **ACO Parameters** | **Values** |
| --- | --- |
| Number of ants | Range of 10-200. |
| Pheromone evaporation rate (ρ) | Range of 0.1-0.5. |
| Pheromone influence (α) | Range of 1-5. |
| Heuristic influence (β) | Range of 1-10. |
| Maximum number of iterations | Range of 10-500. |

**3.5 Cuckoo Search Algorithm**

Cuckoo search optimization is based on the brood parasitism of some cuckoo species. In addition, this algorithm is enhanced by the so-called Lévy flights [21] rather than by simple isotropic random walks. Recent studies show that CSO is potentially far more efficient than PSO and genetic algorithms [11,12,40,41,26].

CSO uses a balanced combination of a local random walk and the global explorative random walk, controlled by a switching parameter. CSO has two distinct advantages over other algorithms such as GA and Simulated Annealing: efficient random walks and balanced mixing. Since Lévy flights are usually far more efficient than any other random-walk-based randomization techniques, CSO can be very efficient in global search. In fact, recent studies show that CSO can have guaranteed global convergence [27]. In addition, the similarity between eggs can produce better new solutions, which is essentially fitness-proportional generation with a good mixing ability. In other words, CSO has varying mutation realized by Lévy flights, and the fitness-proportional generation of new solutions based on similarity provides a subtle form of crossover. A flowchart of its working principle is demonstrated in Figure 3.5s.

**
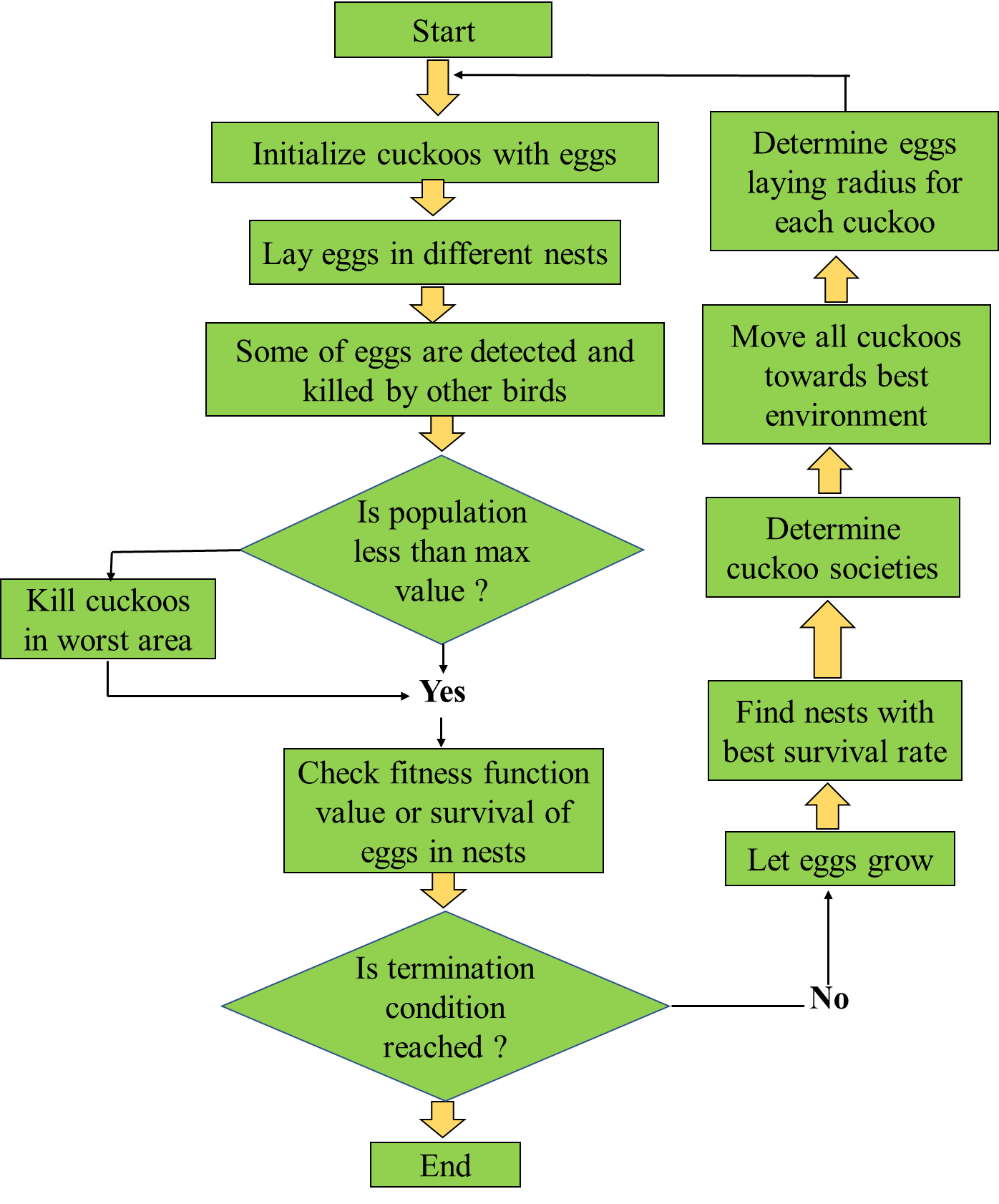
**

**Figure 3.5s.** Flowchart of working principle of Cuckoo Search Algorithm.

| **CSO Parameters** | **Values** |
| --- | --- |
| Population Size (n) | Range of 500-5000 |
| Discovery Rate of Alien Eggs/Probability of Abandonment (pa) | Range of **0.1 to 0.3** |
| Maximum Number of Generations/Iterations | Range of 100-1000 |
| Step Size Control Parameter (α) | Range of **0.01 to 1** |
| Lévy Flight Distribution | **1.5** |

**References**

1. DATA, M.M.P., *Aluminum, Al.* MatWeb, 1996-2023.

2. Gao, Z., et al., *Effects of minor Zr and Er on microstructure and mechanical properties of pure aluminum.* Materials Science and Engineering: A, 2013. **580**: p. 92-98.

3. Rocha, S.S.d., et al., *Vickers hardness of cast commercially pure titanium and Ti-6Al-4V alloy submitted to heat treatments.* Brazilian dental journal, 2006. **17**: p. 126-129.

4. Niinomi, M., *Mechanical properties of biomedical titanium alloys.* Materials Science and Engineering: A, 1998. **243**(1-2): p. 231-236.

5. DATA, M.M.P., *Tungsten, W.* MatWeb 1996-2023

6. Encyclopedia, W.T.F., *Hardnesses of the elements (data page).* Wikimedia Foundation, Inc., 14 September 2023.

7. Technologies, S.S., *Commercially pure nickel 270 (high purity nickel).* SubsTech, 2012/06/03.

8. Holzwarth, U. and H. Stamm, *Mechanical and thermomechanical properties of commercially pure chromium and chromium alloys.* Journal of Nuclear Materials, 2002. **300**(2-3): p. 161-177.

9. DATA, M.M.P., *Tantalum, UNS R05400.* MatWeb, 1996-2023

10. DATA, M.M.P., *Copper, Cu; Annealed.* MatWeb, 1996-2023

11. Cleaves, H.E. and J. Hiegel, *Properties of high-purity iron.* J. Res. Natl. Bur. Standards, 1942. **28**(643): p. 1471.

12. Betteridge, W., *The properties of metallic cobalt.* Progress in Materials Science, 1980. **24**: p. 51-142.

13. DATA, M.M.P., *Vanadium, V; Vacuum Annealed Sheet.* MatWeb, 1996-2023

14. DATA, M.M.P., *Niobium, Nb (Columbium, Cb); Wrought.* MatWeb, 1996-2023

15. MATERIALS, A., *Niobium (UNS R04210).* AZO Nov 15 2012.

16. DATA, M.M.P., *Zinc, Zn.* MatWeb, 1996-2023

17. Tong, X., et al., *Microstructure, mechanical properties, biocompatibility, and in vitro corrosion and degradation behavior of a new Zn–5Ge alloy for biodegradable implant materials.* Acta biomaterialia, 2018. **82**: p. 197-204.

18. Fu, Z., et al., *Superior mechanical properties of pure Zr with heterogeneous structure.* Materials Characterization, 2022. **194**: p. 112437.
